# Sender–receiver subdivisions of the default mode network in perceptual and memory-guided cognition

**DOI:** 10.1073/pnas.2528851123

**Published:** 2026-04-07

**Authors:** Meichao Zhang, Casey Paquola, Katya Krieger-Redwood, Brontë Mckeown, Charlotte Murphy, Chang Liu, Daniel S. Margulies, Robert Leech, Jonathan Smallwood, Elizabeth Jefferies

**Affiliations:** ^a^State Key Laboratory of Cognitive Science and Mental Health, Institute of Psychology, Chinese Academy of Sciences, Beijing 100101, China; ^b^Department of Psychology, University of Chinese Academy of Sciences, Beijing 100049, China; ^c^Department of Psychology, University of York, Heslington, York YO10 5DD, United Kingdom; ^d^Institute for Neuroscience and Medicine, Forschungszentrum Juelich, Juelich 52425, Germany; ^e^Department of Psychology, Queen’s University, Kingston, ON K7L 3N6, Canada; ^f^Integrative Neuroscience and Cognition Centre, Centre National de la Recherche Scientifique and Université de Paris, Paris 75006, France; ^g^Institute of Psychiatry, Psychology & Neuroscience, King‘s College London, London SE5 8AF, United Kingdom

**Keywords:** default mode network, memory, perception, functional connectivity, internal-external attention

## Abstract

Everyday cognition depends on the brain’s capacity to shift between sensing the external world and constructing it from memory. To achieve this, large-scale cortical systems must flexibly integrate incoming sensory signals with internally generated representations. Here, we show that this flexibility is reflected in the macroscale architecture of the default mode network (DMN). Using convergent analyses across three independent fMRI datasets spanning directional connectivity, intrinsic organization, and task-evoked responses, we identify spatially distinct DMN subregions that are preferentially engaged during perceptual decisions about faces or memory-guided decisions based on previously seen images. These subregions correspond to a microarchitectural distinction, captured via directional and intrinsic connectivity profiles: regions preferentially engaged during face perception align with receiver-like, afferent-biased zones that show strong intrinsic connectivity across the heteromodal cortex, a profile that might support information integration during perceptually guided decision-making. In contrast, memory-guided, perceptually decoupled decisions differentially engage sender-like, efferent-biased zones that show broader connectivity with perceptual-motor and attentional systems beyond the DMN. This double dissociation demonstrates a systematic association between DMN connectivity and engagement during perceptually coupled versus memory-guided cognitive processes, providing an organizational account of how DMN architecture relates to flexible human thought.

## Significance

Human cognition depends on flexibly shifting between perception and memory-based thought. These modes place opposing demands on neural systems, consistent with opposite directions of information flow. We show that this flexibility is associated with structural and functional differentiation within the default mode network (DMN), a distributed set of interconnected brain regions active during internally focused thoughts. Distinct subregions are differentially engaged during perceptually grounded versus memory-guided decisions, corresponding to differences in microarchitecture and connectivity. Receiver-like regions, biased toward afferent input, show stronger heteromodal connectivity, whereas sender-like regions, biased toward efferent projections, show stronger coupling with distributed sensorimotor systems. This work offers an organizational framework linking DMN architecture with internal and external cognition, providing insight into flexible human thought.

**Fig. 1.**
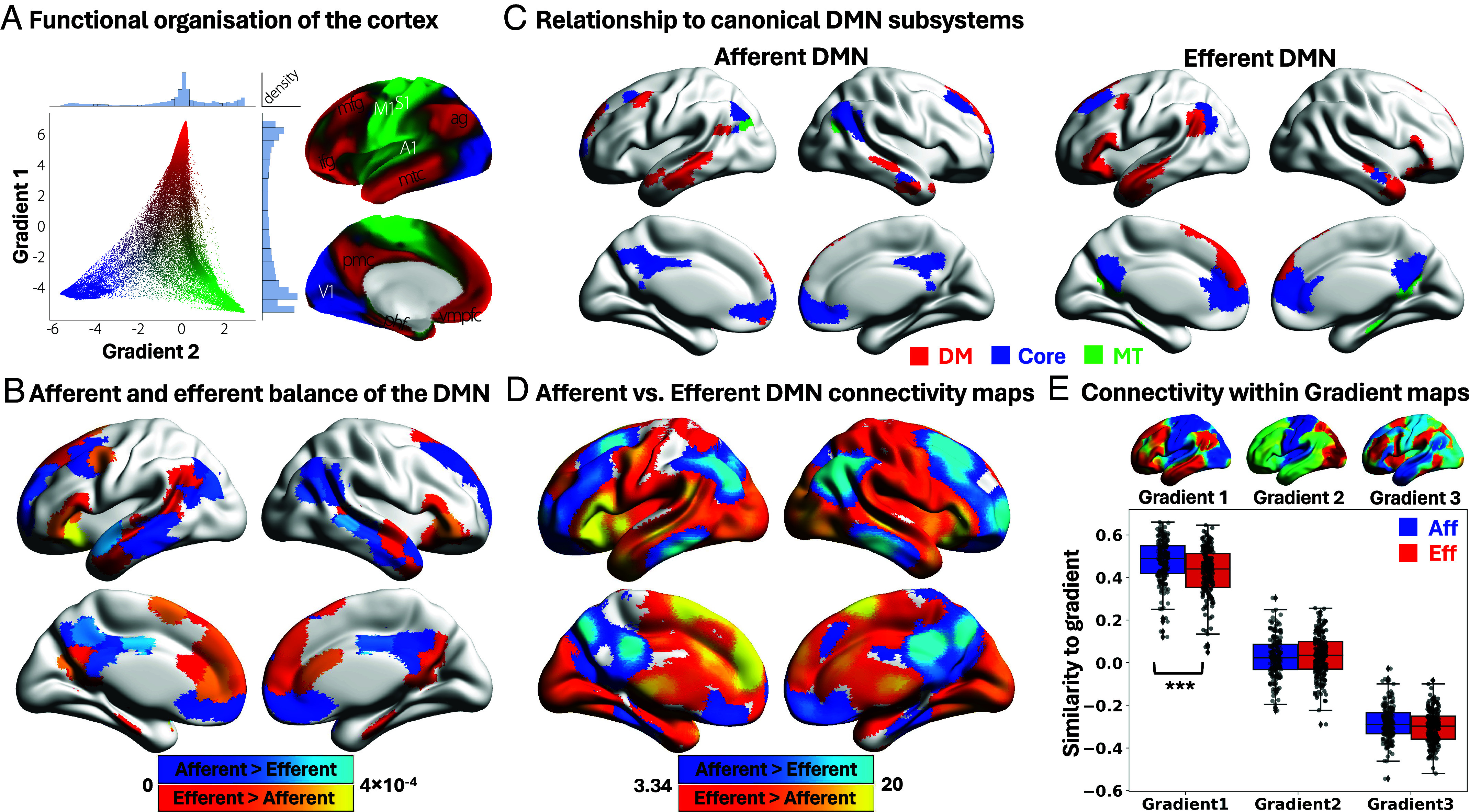
Microarchitecture and functional organization of the DMN. (*A*) Scatterplot of the first two gradients—Gradient 1 (unimodal–transmodal axis), and Gradient 2 (sensorimotor–visual axis). Histograms along each axis display the distribution of gradient values. Colors from the scatterplot are presented on the cortical surface for anatomical orientation. The principal gradient of connectivity spans unimodal sensorimotor (green) and visual (blue) regions to transmodal regions (red), corresponding to the DMN. DMN hubs are distributed across the brain, and functionally most distant from primary sensorimotor systems (7, 8). Reprinted with permission from Margulies et al. (7). (*B*) Contrast of afferent versus efferent connectivity: warm colors = stronger DMN output to non-DMN regions; cool colors = stronger input from other cortices. (*C*) Alignment of afferent/efferent DMN subdivisions with canonical DMN subsystems as described by Yeo et al. (24). Red = dorsomedial (DM) subsystem, blue = core subsystem, and green = medial temporal (MT) subsystem. (*D*) Resting-state functional connectivity of afferent- and efferent-biased DMN subunits: warm colors = stronger connectivity to efferent DMN seed; cool colors = stronger connectivity to afferent DMN seed. (*E*) Distribution of voxel-wise correlations between connectivity maps and cortical gradients. Boxes show the interquartile range; whiskers = 1.5 × IQR; horizontal lines = median. ***P < 0.001. A1, primary auditory; ag, angular gyrus; ifg, inferior frontal gyrus; M1, primary motor; mfg, middle frontal gyrus; mtc, middle temporal cortex; phf, para-hippocampal formation; pmc, posteromedial cortex; S1, primary somatosensory; V1, primary visual; vmpfc, ventromedial prefrontal cortex.

Human cognition flexibly alternates between perceptually coupled states—in which sensory input guides understanding and action, and decoupled states—in which memory retrieval and imagination unfold independently of the immediate environment (1–6). These contrasting modes require neural systems to balance the processing of external inputs with the internal generation and dissemination of memory-based information, yet it remains unclear how the brain achieves this balance without interference. One influential hypothesis proposes that the topographical location of the heteromodal cortex is well placed to be engaged across both states. The default mode network (DMN) is both distant from, and positioned between auditory-motor and visual systems concerned with action and perception [Fig. 1*A*; (7, 8)]. This distance from sensory input has been proposed to align with perceptually decoupled mental states that are relatively independent of sensory input, such as scene construction, episodic simulation, and memory-guided decision-making (1, 3, 9–11). Importantly, the DMN can remain engaged during cognitively demanding tasks that draw on memory, including rule application during task switching or behavioral selection, and when task-relevant information is maintained in working memory rather than derived from ongoing perception (3, 11–15). At the same time, the DMN is also engaged during perceptually rich contexts, such as face and scene processing (16–19), and its topography may support the integration of experiential features into heteromodal representations that underpin spatial navigation, social reasoning, and semantic understanding (1, 16, 20–23). These observations raise fundamental questions about how the heteromodal cortex organizes these divergent cognitive functions.

Gradient-based models of brain organization provide a promising framework for understanding the DMN’s complex functional and structural architecture. Whole-brain intrinsic connectivity gradients delineate continuous axes of cortical function (7), with the DMN anchored at the apex of the unimodal-to-transmodal gradient (Gradient 1), positioned between visual and motor systems on Gradient 2, and largely separated from executive control regions along Gradient 3. When plotted in gradient space (Fig. 1*A*), the DMN’s distance from both primary systems suggests this network may be positioned in a way that is suited to integrating inputs or to broadcasting memory-based outputs to the wider brain, thereby shaping ongoing cognition and behavior. In this way, gradients provide a low-dimensional embedding that situates brain regions and networks according to their connectivity profiles and functional roles (25).

Despite these advances, it remains unclear how the brain’s gradient architecture maps on to transitions between perceptually coupled and decoupled cognition, because few studies have manipulated both cognitive modes within the same paradigm. One possibility is that these modes align with classical functional subdivisions of the DMN, derived from parcellations of intrinsic connectivity (24). Research has linked the core DMN subsystem, including the medial prefrontal cortex and posterior cingulate cortex, to internally focused cognition such as autobiographical memory; in contrast, the dorsomedial subsystem, encompassing left temporal and inferior frontal cortices, has been associated with semantic and social contexts that are often more tightly coupled to perceptual input (1, 26, 27). Although these findings suggest well-defined DMN subnetworks might dissociate across perceptually coupled and decoupled cognition, other studies report recruitment of both subsystems across these contexts (3, 28–30). Alternatively, recent microstructural work suggests that the DMN may be organized along an orthogonal axis defined by differential afferent and efferent connectivity (31), implying distinct “receiver” and “sender” roles that could underpin externally versus internally oriented cognition. Determining whether these cognitive modes map onto functional or microstructural subdivisions of the DMN is critical for understanding how its architecture is organized in a way consistent with flexible transitions between mental states.

**Fig. 2.**
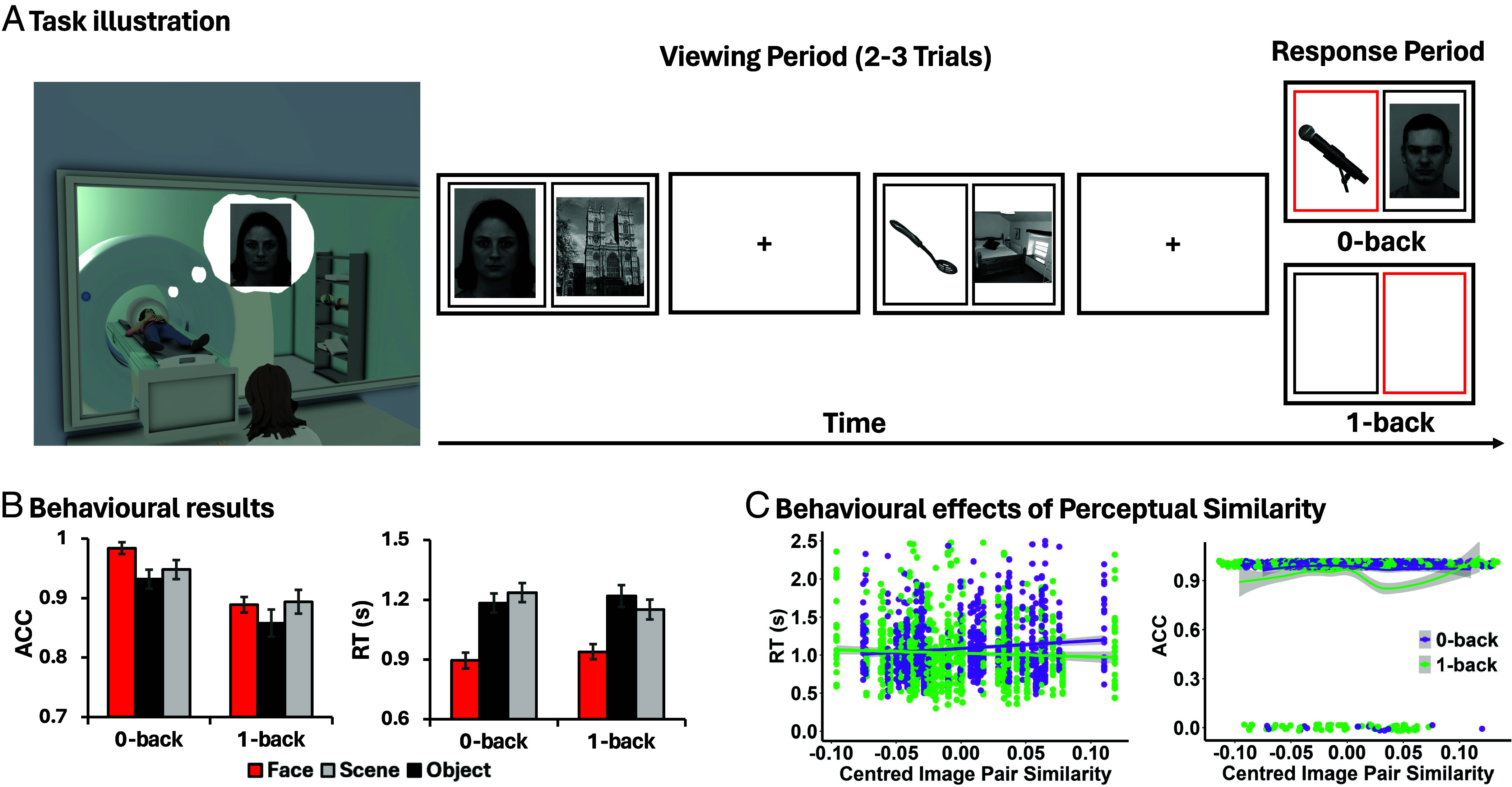
Task illustration and Behavioral results. (*A*) In an adapted n-back task, participants judged the category of stimuli (Face, Scene, and Object) during fMRI. On the 0-back decision-trial, they indicated the category of the image inside a red box (perceptually coupled decision-making). On the 1-back decision-trial, they indicated the category of the image shown in the red box location on the previous trial (memory-guided decision-making). (*B*) The bar charts present the accuracy (ACC; *Left* panel) and response time (RT; in seconds, *Right* panel) for each experimental condition (0-back face/scene/object; 1-back face/scene/object). Error bars denote the SE. (*C*) Behavioral effects of perceptual similarity on 0-back (purple) and 1-back (green) performance. RT is shown with individual trials as points and fitted linear trends (95% CI). Correct and incorrect responses are plotted as jittered points with LOESS (Locally Estimated Scatterplot Smoothing) trend lines for correct trials. Note. Permission to reuse or reproduce the face images must be obtained from the right holders and the Karolinska Directed Emotional Faces (KDEF) (35). The IDs of the KDEF images shown here are AF01NES and AM07NES.

To adjudicate between functional and microstructural accounts of DMN organization, we first characterized its microarchitecture in terms of preferential afferent (input) versus efferent (output) connectivity. Following the framework of Paquola et al. (31), this analysis was applied to an open-access resting-state fMRI dataset (N = 40) (32). We then examined whether this microarchitectural distinction, captured via directional connectivity profiles, aligned with intrinsic functional organization in a second independent resting-state dataset (N = 191). To test whether these features are associated with DMN engagement during perceptually grounded versus memory-guided cognition, we collected task-based fMRI in a third sample (N = 28). Participants performed an adapted n-back task that manipulated both stimulus category (faces, objects, scenes) and decision mode (0-back vs. 1-back; Fig. 2*A*). These categories were chosen based on prior evidence that they differentially modulate DMN responses (16, 19, 26, 33, 34). In the 0-back condition, decisions were about the currently visible stimulus, whereas in the 1-back condition, decisions were based on memory for the stimulus presented at the same spatial location on the previous trial, in the absence of concurrent visual input. Unlike a standard n-back task—which emphasizes continuous working-memory updating and perceptual-memory comparison—our adapted 1-back paradigm was designed to elicit explicit memory retrieval rather than ongoing monitoring. Participants retrieved categorical and spatial information about the previous display while viewing an empty decision frame, without simultaneously encoding new sensory input. Consequently, our 1-back task reduces working-memory load relative to classic n-back designs while relying on internally generated information to guide behavior. By minimizing perceptual input during 1-back decisions, our task provides a clearer behavioral probe of memory-guided behavior, and is therefore well-suited to identifying DMN regions preferentially involved in perceptual versus memory-based cognition. We then tested whether these functional distinctions aligned with afferent- and efferent-biased subunits of the DMN, or instead aligned with functional subdivisions previously defined by intrinsic connectivity (24). Finally, we used gradient-based embedding (7) to situate these task-driven responses within the broader low-dimensional functional architecture of the cortex, allowing us to determine whether DMN states associated with perceptual coupling versus decoupling states reflect not only local microarchitecture but also broader patterns of large-scale cortical organization. In doing so, we bridge recent anatomical models of DMN microstructure with cognitive task engagement, providing evidence that the DMN’s organization tracks externally and internally oriented modes of thought. Because the present study relies on correlational fMRI measures, our conclusions concern the organization and engagement of DMN subregions across cognitive modes, rather than their causal necessity for behavior. Hypotheses about mechanism or functional contributions remain for future work using perturbation or lesion approaches.

## 1. Results

### 1.1. Microarchitecture and Functional Organization of DMN.

#### 1.1.1. Afferent and efferent balance of DMN.

Emerging microstructural evidence indicates that the DMN comprises regions that are receptive to inputs, as well as regions that are more insulated from broader influences (31), consistent with putative “receiver” and “sender” roles. To test whether this microarchitecture reflects distinct afferent (input) versus efferent (output) biases in DMN, we contrasted cortico-cortical effective connectivity profiles derived from regression dynamic causal modeling (rDCM) (36). Paquola et al. (31) applied whole-cortex rDCM to resting-state fMRI timeseries from 400 parcels in 40 healthy adults (32), yielding a scalable generative model of directed connectivity. Within this framework, DMN parcels were examined both as targets for functional input analyses and as seeds for functional output analyses. The contrast between average afferent (DMN as target) and efferent (DMN as seed) estimates provided an index of regional differences in the balance of effective connectivity across the DMN. As shown in Fig. 1*B*, warm-colored regions exhibited stronger output from the DMN to non-DMN areas, while cool-colored regions showed stronger input from non-DMN areas into the DMN. These analyses reveal a functional subdivision within the DMN: one set of regions can be described as resembling “receivers”, with stronger inputs from non-DMN areas, while another set, more insulated, can be described as more “sender-like”, with greater output to the broader cortex.

#### 1.1.2. Microarchitectural-functional dissociation within the DMN.

Intrinsic functional connectivity analyses have divided DMN into dorsomedial, core, and medial temporal subsystems linked to distinct cognitive functions (24, 26). Some studies suggest that core DMN is relatively isolated from input, while dorsomedial prefrontal and lateral temporal regions contribute more strongly to social and semantic processing (1, 37). We next asked whether the afferent- and efferent-biased DMN subunits identified above map onto these conventional DMN subsystems or reflect a distinct organizational axis. As shown in Fig. 1*C*, both afferent- and efferent-dominant zones spanned all three DMN subsystems. This result indicates that the microarchitectural division that we identified via directional connectivity is orthogonal to canonical DMN parcellations based on intrinsic functional coupling.

#### 1.1.3. Functional connectivity of microarchitectural regions.

The next analysis investigated whether afferent- and efferent-biased DMN subregions show distinct intrinsic coupling patterns along the cortical hierarchy from the sensory-motor to heteromodal cortex. Using an independent dataset (N = 191), we computed whole-brain resting-state functional connectivity with the efferent- and afferent-biased DMN as seeds (Fig. 1*B*). Efferent-biased DMN regions showed stronger connectivity with motor and visual systems (warm colors), while afferent-biased DMN regions were more strongly coupled to heteromodal regions (cool colors; Fig. 1*D*). Connectivity with Yeo-7 networks (with afferent and efferent DMN voxels mutually excluded to avoid circularity in DMN-to-DMN connectivity) showed that efferent-biased regions coupled more strongly with sensory-motor, ventral attention, and limbic networks, whereas afferent-biased regions preferentially connected to dorsal attention, frontoparietal, and efferent DMN regions (*SI Appendix*, Supplementary Analysis 1).

To situate these effects within the brain’s macroscale functional architecture, we embedded these connectivity profiles within the brain’s low-dimensional functional gradients by correlating afferent/efferent connectivity maps with the first three gradient maps for each participant, excluding seed voxels to avoid bias (Fig. 1*E*). Statistical significance was assessed using nonparametric permutation testing (10,000 iterations), comparing the observed mean difference in spatial similarity against a null distribution, with Bonferroni correction across gradients. A robust difference between afferent and efferent-biased DMN subunits emerged for Gradient 1 (*P* < 0.001), but not for Gradient 2 (*P* = 1) or Gradient 3 (*P* = 0.10; see Fig. 1*E* for results and *SI Appendix*, Fig. S1 for the corresponding null distributions). Afferent-biased DMN regions aligned with the transmodal apex of Gradient 1, whereas efferent-biased DMN regions aligned with its unimodal end. This pattern is consistent with the interpretation that afferent-biased DMN sites are preferentially associated with multisensory integration, whereas efferent-biased DMN regions show broader connectivity across sensorimotor and attentional networks. Together with rDCM, these results indicate a consistent organizational principle: the directed connectivity within DMN (afferent vs. efferent) is aligned with its large-scale functional embedding, linking DMN microarchitecture with hierarchical information flow across the cortex.

### 1.2. Task Behavior.

Building on the observation that afferent- and efferent-biased DMN regions show complementary directed and intrinsic connectivity profiles, we next examined how these subunits respond during perceptually grounded and memory-guided cognition using task-based fMRI. In an adapted n-back task, we manipulated both the mode of decision-making (i.e., perception- vs. memory-guided) and the content of decisions (faces, scenes, and objects), as these stimulus categories differentially modulate DMN responses (e.g., 16, 19, 26, 33, 34). Participants performed category judgments based either on immediate visual input (0-back condition) or short-term memory (1-back condition; Fig. 2*A*). On response trials, a spatial cue highlighted one of two lateralized frames, and participants reported whether the image presented at the cued location was a face, scene, or object. In the 0-back condition, the cued image remained visible at the time of decision, whereas in the 1-back condition no image was shown, and responses relied on participants’ memory of the image presented previously at that location. Trial types were intermixed rather than blocked to minimize differences in encoding strategy across tasks. Although all trials involved visual encoding and short-term maintenance, the critical manipulation concerned the informational basis of the decision. Specifically, 0-back decisions were anchored to concurrent perceptual input, whereas 1-back decisions relied solely on memory since no images were available during the decision period. Thus, the task contrasted perceptually grounded versus memory-guided decisions without assuming full functional decoupling between perceptual and mnemonic systems. Unlike classic n-back paradigms, this design also isolated memory retrieval rather than parametrically manipulating cognitive load, allowing us to identify DMN regions supporting categorical processing in a perceptually coupled state, compared with functional variation in DMN linked to perceptual decoupling.

#### 1.2.1. Behavioral results.

The results are shown in Fig. 2*B*. All post hoc *t* tests were Holm–Bonferroni corrected for the number of comparisons. Repeated-measures ANOVA revealed that 0-back was more accurate than 1-back [*F*(1,23) = 37.83, *P* < 0.001, *η_p_^2^* = 0.62], and there were differences between categories [*F*(2,46) = 6.03, *P* = 0.005, *η_p_^2^* = 0.21], with faces judged more accurately than objects [Face vs. Object: *t*(23) = 3.50, *P* = 0.009; Scene vs. Object: *t*(23) = 2.36, *P* = 0.10; Face vs. Scene: *t*(23) = 1.33, *P* = 0.66]. There was no interaction [*F*(2,46) = 1.90, *p* = 0.16, *η_p_^2^* = 0.08]. For response time (RT), there was no main effect of Task [*F*(1,23) = 0.003, *P* = 0.95, *η_p_^2^* < 0.001], but a strong effect of Category [*F*(2,46) = 69.19, *P* < 0.001, *η_p_^2^* = 0.75] and an interaction [*F*(2,46) = 12.06, *P* < 0.001, *η_p_^2^* = 0.34]. On 0-back trials, face decisions were particularly fast [Face vs. Scene: *t*(23) = −10.33, *P* < 0.001; Face vs. Object: *t*(23) = −7.23, *p* < 0.001; Scene vs. Object: *t*(23) = 1.96, *P* = 0.18). On 1-back trials, faces were again fastest, but scenes were also faster than object [Face vs. Object: *t*(23) = −8, *P* < .001; Face vs. Scene: *t*(23) = −7.61, *P* < 0.001; Scene vs. Object: *t*(23) = −3.19, *P* = 0.012]. These behavioral results suggest that decisions are more demanding when guided by memory than perception, and that face targets are more visually salient than other targets.

#### 1.2.2. Behavioral effects of perceptual similarity.

The next analysis tested the hypothesis that 0-back decisions are more perceptually coupled, and therefore more strongly influenced by the surface characteristics of the images. We examined the extent to which visual similarity between image pairs influenced performance in 0-back versus 1-back conditions. Each stimulus was passed through a pretrained ResNet-50, and feature vectors from a mid-to-high-level layer were used to compute trial-wise pairwise perceptual similarity between image pairs. Linear mixed-effects models were used for RT and generalized linear mixed-effects models (GLMM, logistic link) for accuracy, with full model details in *SI Appendix*, Supplementary Analysis 2. As shown in Fig. 2*C*, RT showed a significant interaction between similarity and task [*F*(1,1589.92) = 12.90, *P* < 0.001], with higher visual similarity eliciting slower responses in 0-back trials [*β* = 1.15, SE = 0.26, *t*(101) = 4.41, *P* < 0.001], but not in 1-back trials [*β* = −0.18, SE = 0.26, *t*(101) = −0.69, *P* = 0.50]. Although the interaction between visual similarity and task was not significant for accuracy (*β* = 6.60, SE = 4.85, *z value* = 1.36, *P* = 0.17), the pattern mirrored the RT results: similarity negatively predicted performance in 0-back trials (*β* = −9.54, *SE* = 4.40, *P* = 0.030) but not 1-back trials (*β* = −2.94, *SE* = 3.26, *P* = 0.173). These findings confirm that 0-back decisions are more strongly driven by perceptual characteristics than 1-back decisions.

### 1.3. Whole-Brain Univariate fMRI Analysis.

We next identified DMN regions sensitive to different cognitive demands. We focused on areas that distinguished i) memory from perceptual judgments (1-back vs. 0-back) and ii) stimulus category during perceptually coupled decisions (faces, scenes, objects in 0-back). This allowed us to isolate DMN responses linked to perceptual decoupling versus sensory content. Category effects were similar across 0-back and 1-back trials (*SI Appendix*, *Supplementary Materials*).

#### 1.3.1. Effects of Task.

**Fig. 3.**
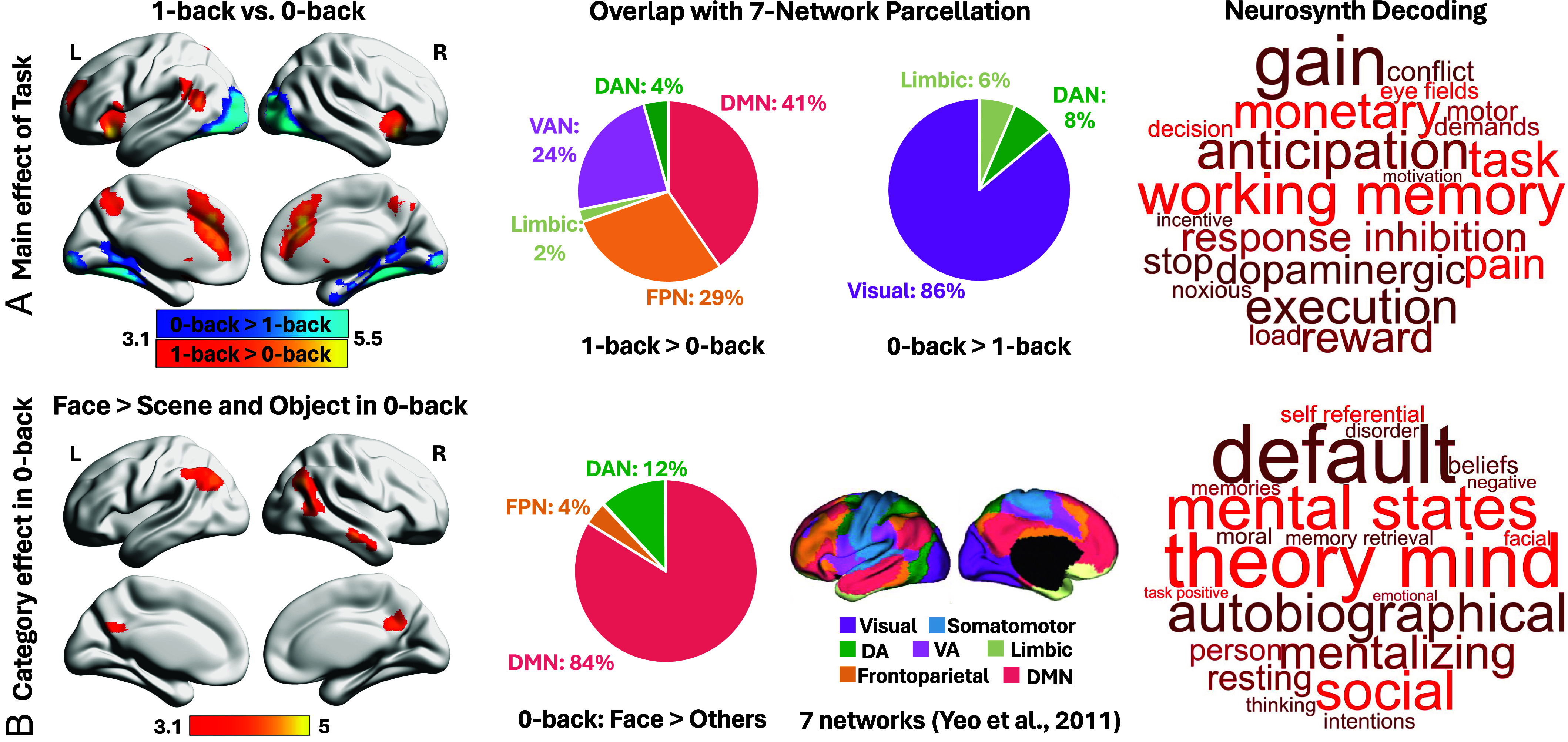
Neural correlates of task mode and stimuli category in 0-back task. (*A*) Regions more active during perceptual decisions (*0-back > 1-back*; blue) or memory-guided decisions (*1-back > 0-back*; red). Pie charts show the proportion of significant voxels associated with each task that fell within each large-scale network defined by Yeo et al. (24) in a 7-network parcellation of whole-brain intrinsic functional connectivity. Using Neurosynth, a meta-analysis of 1-back regions revealed a strong association with the functional terms of “task” and “working memory”. (*B*) Regions responding more strongly to face than scene/object decisions in 0-back task. Many voxels overlapped with the DMN. Neurosynth meta-analysis of face-sensitive regions revealed a strong association with the functional terms of “default”, “theory mind”, and “social”. All maps are thresholded at *z* > 3.1 (*P* < 0.05). DA = Dorsal attention; VA = Ventral attention; DMN = Default mode network; L = Left hemisphere; R = Right hemisphere.

The contrast of *1-back > 0-back* revealed activation in insular and frontal orbital cortex, frontal pole, anterior paracingulate/cingulate cortex, precuneus, and angular gyrus (red regions in Fig. 3*A*). These voxels were concentrated in heteromodal networks, including DMN (41%) and FPN (29%) according to Yeo et al.‘s (24) 7-network parcellation (see pie chart in Fig. 3*A*), in line with prior studies of memory-guided tasks (3, 11, 15). By contrast, *0-back > 1-back* activated occipital pole, lateral occipital cortex, lingual gyrus, precuneus cortex, temporal occipital fusiform, and temporal fusiform cortex (blue regions in Fig. 3*A*), with 86% of voxels falling in the visual network (see pie chart in Fig. 3*A*).

#### 1.3.2. Effects of Category in perceptual decisions.

Face decisions during 0-back elicited stronger responses than scenes and objects in bilateral lateral occipital cortex, angular gyrus, posterior cingulate/precuneus, and right middle temporal gyrus (Fig. 3*B*). Strikingly, 84% of these face-selective voxels fell within DMN (24) (pie chart in Fig. 3*B*). Similar clusters were observed for *Face > Scene/Object* across 0- and 1-back tasks (*SI Appendix*, Fig. S2). In addition, *Scene > Face/Object* in both the 0-back task and across 0- and 1-back tasks (*SI Appendix*, Fig. S2) mainly engaged the visual cortex, while no clusters emerged for *Object > Scene/Face*. Therefore, only face processing reliably recruited DMN.

#### 1.3.3. Dissociation of Task and Category effects in DMN.

DMN responded to both perceptual decoupling (*1-back > 0-back*) and perceptually coupled face decisions (*Face > Scene/Object* in 0-back), but these effects were spatially distinct, demonstrating a functional dissociation. i) No voxels were shared between these two contrasts (*SI Appendix*, Fig. S3), and ii) further ROI analyses in these DMN regions revealed no category effects in the 1-back DMN map and no task effects in the face 0-back DMN map (*SI Appendix*, Fig. S3). Both effects overlapped with the core DMN subsystem in Yeo et al.‘s (24) 17-network parcellation (*SI Appendix*, Fig. S4). Neurosynth meta-analysis further underscored their distinct functional profiles: 1-back clusters were associated with “task” and “working memory”, while face-related clusters were linked to “default”, “theory of mind”, and “social” (see word clouds in Fig. 3).

#### 1.3.4. Category effects in the visual cortex.

Although our focus was on DMN, category-selective responses were robust in visual network (24). The parahippocampal place area responded preferentially to scenes, the fusiform face area to faces, and the lateral occipital cortex to objects [*SI Appendix*, Fig. S5; (38–40)]. These regions also showed Task by Category interactions, with stronger selectivity during 0-back, whereas the absence of category selectivity in the 1-back condition likely reflects reduced reliance on visual input (*SI Appendix*, Fig. S6). Thus, category effects were widespread across the visual cortex but limited within DMN, emerging only for faces.

### 1.4. Spin Tests of DMN’s Topographical Location.

Next we asked whether DMN regions engaged during both memory-guided cognition (*1-back > 0-back*) and perceptually coupled face decisions (*face > other categories in 0-back*) both fall at the transmodal apex of the principal cortical gradient, which spans sensory-motor to heteromodal cortex (7, 8). We also asked whether this transmodal positioning characterizes DMN subregions defined by afferent versus efferent connectivity.

**Fig. 4.**
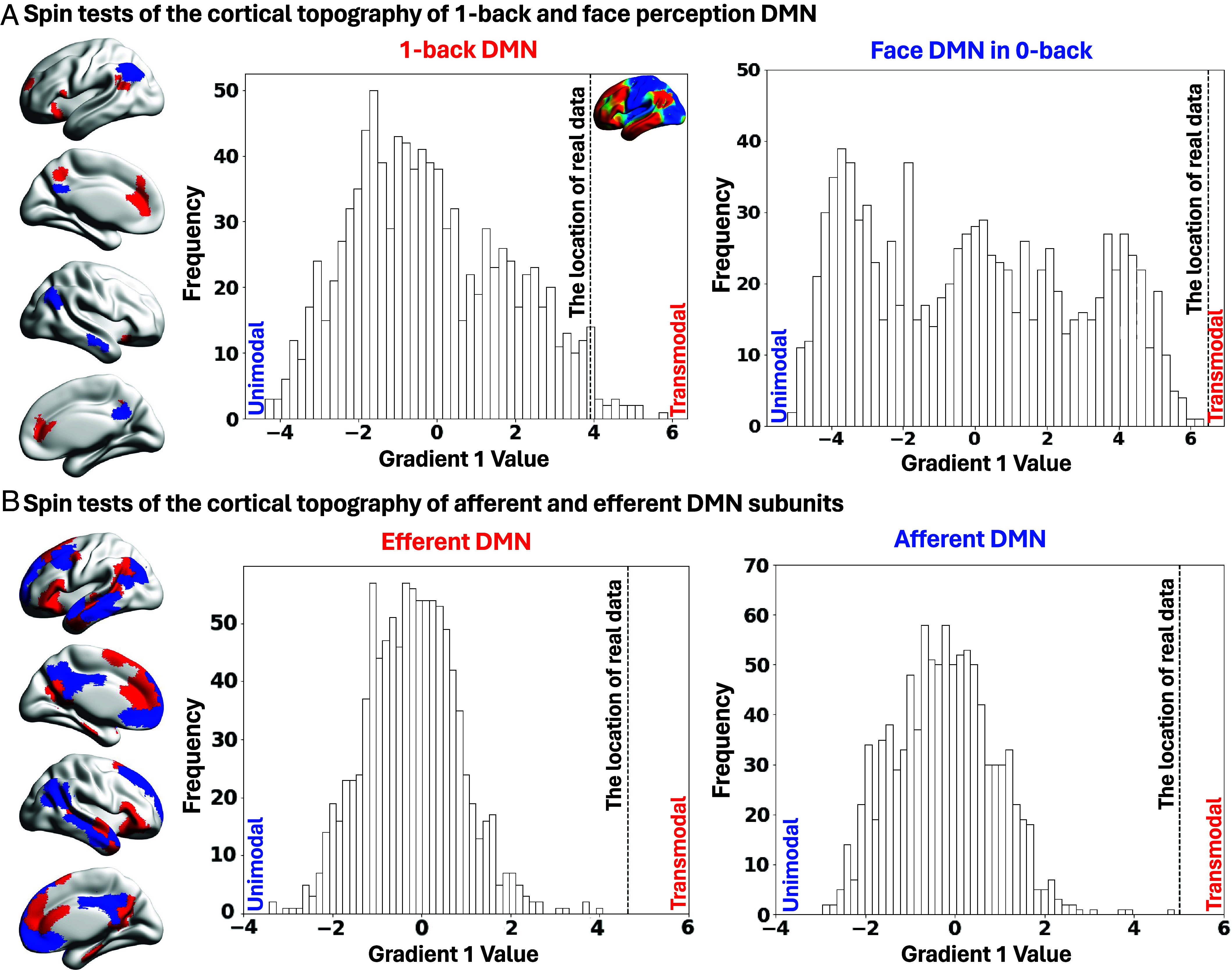
Spin tests of the DMN locations. Panels (*A* and *B*) show the null distribution for DMN regions identified from the contrasts of *1-back > 0-back* and *Face > Scene/Object in 0-back* task, as well as for the afferent and efferent DMN subunits. Gradient 1 (*Top* *Left*) separates transmodal (red) from the unimodal cortex (blue). Histograms depict the frequency distribution of 1,000 spin permutations, with the dashed line indicating the observed location of the actual data on Gradient 1. These results demonstrate that all tested DMN regions occupy the transmodal apex significantly beyond chance.

Using spin permutation tests, we compared the mean Gradient 1 value of each DMN response against a null distribution of average gradient values generated by 1,000 spherical rotations. Both 1-back and face-responsive DMN clusters lay significantly beyond chance expectations on the transmodal end of Gradient 1 (1-back DMN: *P* = 0.038; face-responsive DMN: *P* < 0.001; Bonferroni corrected for two tests; Fig. 4*A*). Likewise, DMN subunits defined by afferent and efferent connectivity profiles also occupied transmodal locations (both *P* < 0.001; Bonferroni corrected for two tests; Fig. 4*B*). Thus, DMN regions engaged during both perceptually coupled and decoupled states, as well as those defined by afferent/efferent connectivity profiles, converge near the transmodal apex of the principal gradient, linking task-elicited responses to microarchitectural subunits along the cortical hierarchy.

Notably, a canonical functional subdivision of DMN, defined by Yeo et al.‘s (24) 17-network parcellation of resting-state fMRI, showed a different pattern. The Core and Dorsomedial DMN subsystems were significantly transmodal (*P* < 0.001 for both), whereas the Medial Temporal subsystem did not differ from chance (*P* = 0.71; Bonferroni corrected; *SI Appendix*, Fig. S7). These findings suggest that while “receiver” and “sender” DMN subregions share a common transmodal location, traditional resting-state subdivisions vary along this axis, implying that the afferent/efferent distinction cuts orthogonally across classical functional boundaries.

### 1.5. Double Dissociation Across Afferent and Efferent DMN Subunits.

**Fig. 5.**
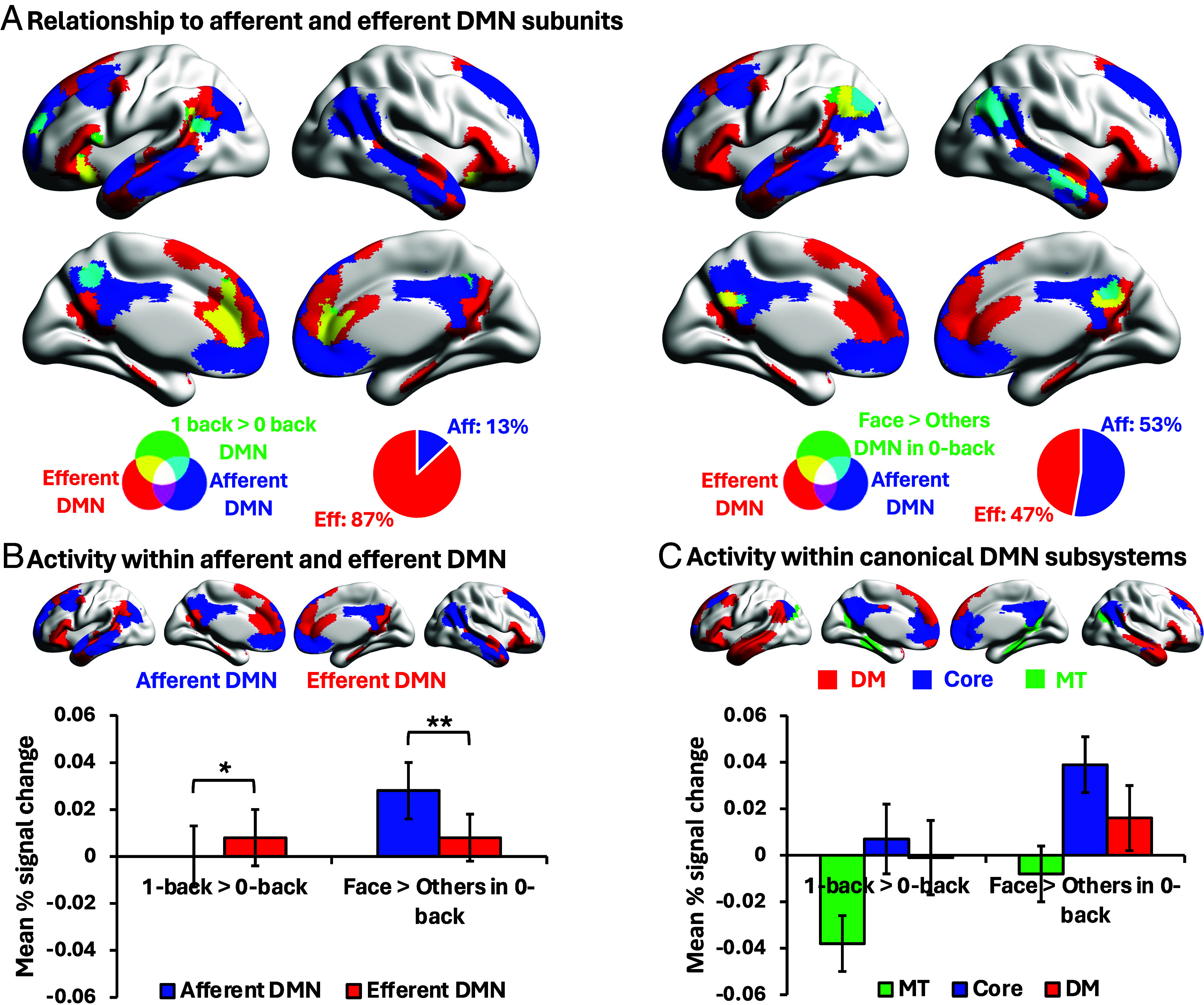
Relationship to afferent/efferent DMN subunits and canonical DMN subsystems. (*A*) Spatial relationship between task-evoked DMN activity (green) and the afferent (blue) and efferent (red) DMN subunits. Yellow voxels indicate overlap with the efferent subunit, and cyan voxels indicate overlap with the afferent subunit. Pie charts show the proportion of significant voxels from each task contrast (*1-back > 0-back*; *Face > Scene/Object in 0-back*) falling within each subunit. (*B* and *C*) Mean percentage (%) signal change extracted from (*B*) afferent/efferent DMN subunit ROIs and (*C*) DMN subsystems ROIs defined by Yeo et al. (24) under the task contexts of *1-back > 0-back* and *Face > Scene/Object in 0-back*. Error bars depict the SEM. *P value < 0.05. **P value < 0.01.

Having established that both task-evoked and microarchitectural subdivisions of the DMN converge near the transmodal apex of the cortical hierarchy, we next asked whether afferent and efferent subunits—or alternatively, the canonical functional subdivisions previously defined by intrinsic connectivity (24)—could capture the functional dissociation between perceptually grounded and memory-guided cognition in task fMRI. We first examined the spatial overlap of DMN regions implicated in perceptually coupled face judgments (*Face > Scene/Object in 0-back*) versus memory-guided cognition (*1-back > 0-back*) with the afferent- and efferent-biased DMN subunits (Fig. 5*A*). The majority of DMN voxels recruited during the 1-back task fell within the efferent subunit (87%), whereas face-responsive DMN regions in 0-back task were distributed across afferent (53%) and efferent (47%) subunits.

Next, we extracted mean percentage signal change within afferent and efferent DMN masks for both contrasts in each participant. A 2 (DMN subunit: afferent vs. efferent) by 2 (Task Contrast: *1-back > 0-back* vs. *Face > Scene/Object in 0-back*) repeated-measures ANOVA revealed a main effect of DMN subunit, *F*(1,23) = 4.79, *P* = 0.039, *η_p_^2^* = 0.17, but not Task Contrast, *F*(1,23) = 1.13, *P* = 0.30, *η_p_^2^* = 0.05. Critically, the interaction was significant, *F*(1,23) = 14.55, *P* < 0.001, *η_p_^2^* = 0.39. Post hoc *t*-tests revealed a stronger response in the efferent than afferent-biased DMN for the task context of *1-back > 0-back* [*t*(23) = 2.00, *P* = 0.028], alongside stronger recruitment of the afferent than efferent-biased DMN for face decisions in 0-back [*t*(23) = 4.00, *P* = 0.001, Fig. 5*B*). This analysis reveals a functional double dissociation between afferent and efferent DMN subregions.

A parallel analysis tested whether a similar dissociation was evident within canonical DMN subsystems defined by Yeo et al.‘s (24) 17-network parcellation (Medial Temporal, Core, and Dorsomedial DMN). A 2 (Task) × 3 (Subsystem) ANOVA revealed a main effect of subsystem, *F*(2,46) = 16.24, *P* < 0.001, *η_p_^2^* = 0.41, but no main effect of Task Contrast, *F*(1,23) = 3.42, *P* = 0.08, *η_p_^2^* = 0.13, and no interaction, *F*(2,46) = 0.74, *P* = 0.48, *η_p_^2^* = 0.031 (Fig. 5*C*). Thus, unlike the afferent/efferent subdivision, canonical DMN subsystems did not exhibit a functional specialization for perceptual coupling versus decoupling.

Together, these findings demonstrate that afferent and efferent DMN subunits track a robust cross-over pattern of task engagement. Efferent-biased DMN regions show stronger engagement during memory-guided, perceptually decoupled decisions, whereas afferent-biased DMN regions respond more strongly during perceptually coupled face decisions, consistent with greater association with high-level visual input. Since this double dissociation was not captured by conventional DMN subsystems, effective connectivity-defined DMN subdivisions provide a useful organizational description of how different task demands recruit the DMN.

### 1.6. Whole-Brain Activity Patterns Decomposed Using Functional Connectivity Gradients.

**Fig. 6.**
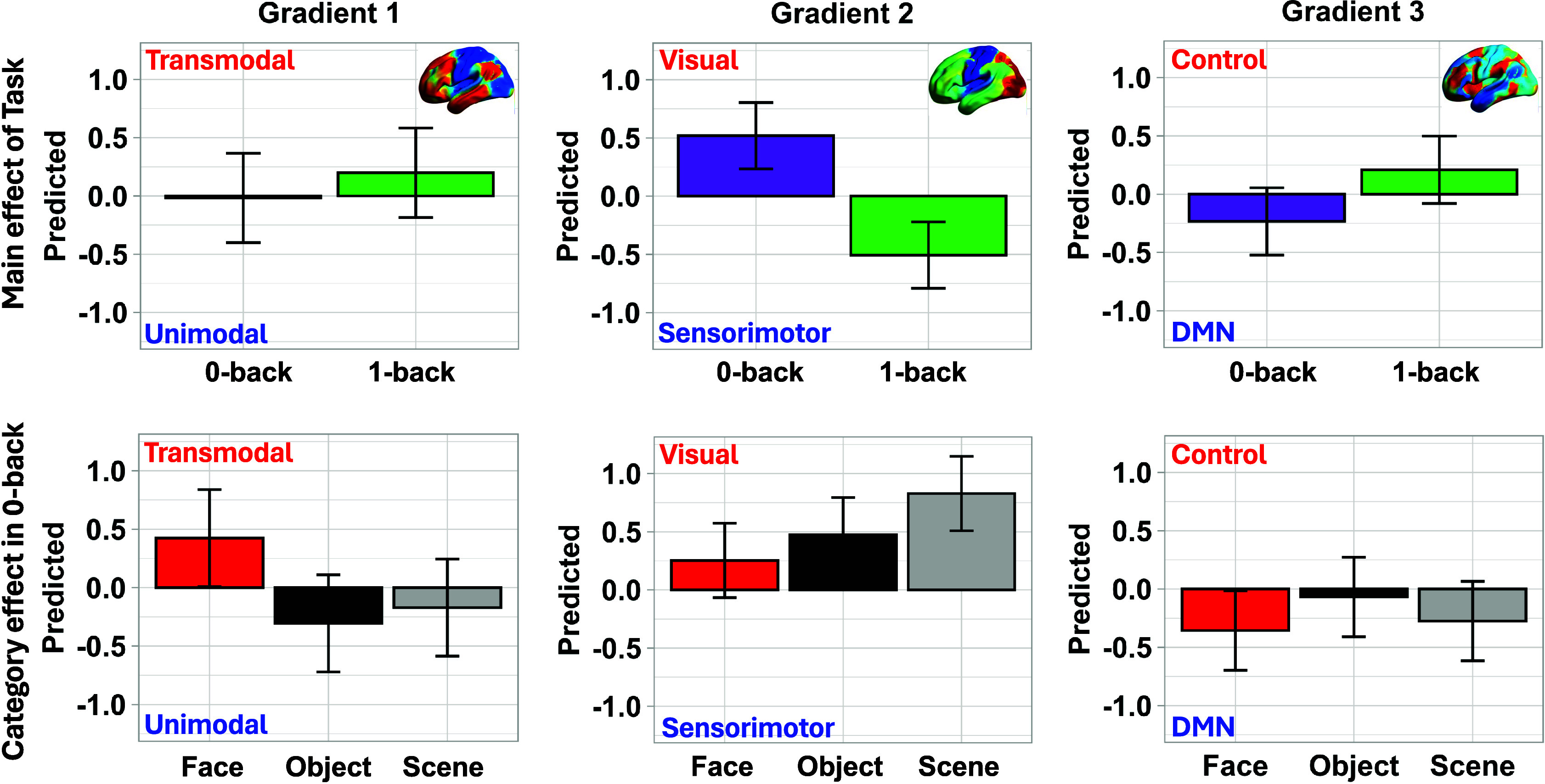
State-space analyses of task-evoked activation patterns. Spatial similarity was computed between task-evoked activation maps and the first three gradients of functional connectivity described by Margulies et al. (7). Bar charts show estimated marginal means (predicted means from the linear mixed-effects models) for differences by task mode (1-back vs. 0-back) and by content in 0-back (Face vs. Object vs. Scene). Panels correspond to the first three principal gradients of cortical organization: (*Left*) Gradient 1, transmodal-unimodal; (*Middle*) Gradient 2, visual-motor; (*Right*) Gradient 3, control-DMN. Error bars denote 95% CI.

Thus far, we have demonstrated a functional dissociation within DMN, with distinct subdivisions preferentially engaged by perceptual decoupling and by face perception. While these responses localize to different microarchitectural zones of DMN, they may also reflect broader differences in the brain’s global functional architecture. Specifically, these DMN states might be associated with different patterns of activity across primary systems and control-related regions. To test this, we assessed how task-evoked activation patterns align with the first three connectivity gradients from Margulies et al. (7) (Fig. 6), by computing spatial similarity between activation maps and each gradient. Gradient 1 captures the axis from the unimodal to transmodal cortex and is often interpreted as a proxy for DMN involvement. Gradient 2 spans a visual-to-motor continuum, providing a test of whether perceptually decoupled thought is associated with reduced visual input and increased motor engagement. Gradient 3 distinguishes control-related from automatic processing within the heteromodal cortex, and thus may help separate the greater task demands of 1-back memory judgments from the more automatic processing of faces during 0-back. We hypothesized that both perceptually decoupled (1-back) and face-related (0-back) states would be situated toward the transmodal apex of Gradient 1, consistent with DMN engagement, but would diverge along Gradients 2 and 3 due to differences in sensory coupling and cognitive control.

Linear mixed effects models were examined separately for each gradient, with fixed effects of Task (0-back vs. 1-back), Category (Face, Scene, Object), and their interaction. Full model results are reported in Supplementary Analysis 9; here we highlight the effects most relevant to perceptual decoupling and face processing (Fig. 6). For Gradient 1 (unimodal–transmodal axis), both 1-back and face perception elicited activation toward the transmodal end, consistent with DMN recruitment. Activation was more transmodal in 1-back than in 0-back [*b* = –0.22, 95% CI [–0.39, –0.05], *t*(115) = –2.53, *P* = 0.013]. Within 0-back, faces were more transmodal than objects [*b* = 0.73, 95% CI (0.33, 1.13), *t*(115) = 4.93, *P* < 0.001] and scenes [*b* = 0.60, 95% CI (0.20, 0.99), *t*(115) = 4.02, *P* = 0.001]. For Gradient 2 (visual-to-motor axis), 1-back showed stronger motor alignment relative to 0-back [*b* = 1.03, 95% CI (0.87, 1.18), *t*(115) = 13.25, *P* < .001], consistent with reduced reliance on visual input in this decoupled state. In contrast, face decisions in 0-back occupied a midpoint, being less visually anchored than scenes [*b* = –0.58, 95% CI (–0.94, –0.22), *t*(115) = –4.29, *P* = 0.001]. For Gradient 3 (control–automatic axis), 1-back was more control-related than 0-back [*b* = –0.44, 95% CI (–0.63, –0.26), *t*(115) = –4.67, *P* < 0.001], while no category differences emerged in 0-back.

In conclusion, both perceptually decoupled decisions and face perception engaged transmodal DMN regions, but within distinct whole-brain configurations. The 1-back task is less visually grounded and more controlled, while face perception involves more balanced sensory engagement and more automatic processing. These patterns partly echo the DMN’s microarchitecture: afferent-biased regions, active during face perception, connect with heteromodal DMN and frontoparietal networks that are associated with the balance of visual and motor features, whereas efferent-biased regions engaged during 1-back task link more strongly to sensory-motor systems, consistent with the dissemination of internally generated information across sensorimotor regions. Yet these whole-brain patterns are not reducible to intrinsic connectivity. For instance, efferent-DMN regions recruited by 1-back decisions are not disproportionately connected to control networks, despite this task’s higher control demands. Thus, recruitment reflects task-driven reconfiguration rather than fixed network structures.

## 2. Discussion

How the brain flexibly alternates between internally and externally oriented cognition remains a central question in systems neuroscience. By integrating convergent analyses across three independent datasets—spanning directional connectivity, intrinsic organization, and task-evoked responses—we identify a systematic relationship between a microarchitectural axis and task engagement during perceptually grounded versus memory-guided decisions. We demonstrate that a distinction in directional connectivity, between afferent- and efferent-biased DMN regions, aligns with a cognitive dissociation. Afferent-biased subregions are preferentially engaged during perceptually grounded face decisions and exhibit stronger heteromodal connections, whereas efferent-biased subregions are preferentially recruited during perceptually decoupled, memory-guided decisions, and show broader sensory-motor connections. These observations motivate the hypothesis that afferent-biased subregions have receiver-like connectivity profiles consistent with engagement in contexts requiring the integration of sensory inputs with higher-order representations, whereas efferent-biased subregions have sender-like connectivity profiles consistent with engagement in contexts requiring internally generated information to be coordinated with distributed sensorimotor systems. This double dissociation indicates that DMN engagement across external and internal cognition is not uniform but follows its microarchitectural differentiation into complementary receiver- and sender-like zones.

These microarchitectural and functional differences are embedded within the broader topography of cortical organization. Situated at the transmodal apex of the brain’s principal connectivity gradient, the DMN lies between unimodal visual and motor systems. Its cortical position is consistent with its engagement during both memory-based, decoupled cognition, and perceptually grounded tasks that integrate sensory input with heteromodal representations for semantic and social understanding (8). Thus, the DMN’s flexible response across diverse forms of thought may reflect a convergence between differentiation in its microarchitecture and its privileged location in large-scale functional connectivity space. While prior accounts emphasized its apex position (7), our findings show that afferent–efferent connectivity differences provide an additional organizational dimension associated with its divergent cognitive roles, thereby linking theoretical accounts of DMN function to anatomical specialization. Importantly, the functional divergence described here was not evident in canonical DMN parcellations (24), suggesting that effective connectivity-based microarchitecture describes aspects of functional differentiation not captured by earlier work.

Placing these findings within the brain’s macroscale gradients highlights how local DMN microarchitecture is embedded in global cortical organization. Both perceptually grounded and memory-guided states recruited the transmodal apex of the principal gradient, consistent with DMN’s role in high-level cognition. Yet these whole-brain states diverged along secondary gradients: face perception showed balanced recruitment of visual and motor systems, whereas memory-guided decisions were more control-aligned and biased toward the motor end of the visual–motor gradient, mirroring the efferent profile of these regions. Importantly, this shows that the same DMN regions can participate in distinct functional networks over time, maintaining different configurations depending on task demands. This gradient-based embedding illustrates how DMN subregions participate in distinct large-scale configurations across cognitive requirements, in line with their sender–receiver connectivity profiles. Overall, our findings reinforce that the DMN is not a unitary system but a topographically and functionally differentiated network that shows systematic variation in engagement across cognitive modes (8).

These findings also refine predictive processing models, which propose that the DMN operates as a top–down, generative system projecting high-level predictions to constrain sensory processing (41–43). Our results are consistent with the possibility that predictive and integrative roles are differentially associated with DMN subregions: efferent (sender) regions may be preferentially engaged during contexts consistent with top–down signaling, whereas afferent (receiver) regions may be preferentially responsive during contexts requiring integration of bottom–up inputs. These architectural differences may relate to the DMN’s versatility across cognitive domains, such as episodic stimulation, semantic cognition, and social reasoning, where both the generation and integration of information are required (5).

Important questions remain regarding the cognitive characteristics of this functional dissociation. We use the term “double dissociation” descriptively, to indicate a crossover pattern of task engagement across DMN subregions, rather than as evidence for independent causal modules (44). These organizational distinctions generate testable hypotheses about the potential causal contributions of afferent- and efferent-biased DMN subregions to perceptually coupled and decoupled cognition, which could be further investigated using methods capable of establishing causal relationships, such as brain stimulation, neuropsychology, or studies examining age-related changes in DMN function. Task difficulty is another important consideration, as DMN typically deactivates under demanding contexts. In our data, afferent-biased regions responded more strongly to faces—the easiest categorization condition—whereas efferent-biased regions were engaged during more demanding memory-guided decisions, consistent with prior studies (3, 11, 15). This pattern cannot be explained by a unitary “task-negative” account of DMN, though future work is needed to clarify how afferent and efferent DMN subsystems interact with diverse task demands. Notably, however, face-selective responses in afferent-biased regions appear to reflect specific information-processing demands rather than task ease, as these regions did not show differential deactivation in harder conditions, such as 1-back trials or 1-back object decisions (see *SI Appendix*, Supplementary Analysis 4). Moreover, while our adapted 1-back task was designed to isolate memory-guided decisions from concurrent perceptual input, this decoupling does not imply that perceptual and mnemonic systems operate independently in this condition. Both 0-back and 1-back involve visual encoding, short-term maintenance, and decision operations, which may occur serially, in parallel, or with dynamic coupling. Our manipulation isolates the information available at the decision point, but cannot resolve the precise temporal interplay between perceptual and memory computations. Future studies could probe information flow between these systems under different cognitive states. Finally, it remains an open question whether these regions form part of a third visual-to-DMN pathway (45) for social and semantic cognition [cf. (46)], or instead integrate a broader range of salient perceptual features. In either case, the findings suggest that afferent DMN regions are preferentially engaged during contexts involving meaningful perceptual inputs alongside memory-based processing, particularly for behaviorally relevant stimuli such as faces.

Despite these open questions, the present findings reveal a striking functional double dissociation within the DMN: afferent-biased regions are preferentially engaged during perceptually coupled decisions about faces, while efferent-biased regions are preferentially engaged during perceptually decoupled, memory-guided decisions. These cognitive modes are associated with distinct patterns of functional embedding with primary systems—balanced visual-motor recruitment for face-related DMN zones versus visual decoupling for memory-related zones, aligned with efferent connectivity. This dissociation highlights how gradient-based embedding can uncover principled functional divisions within the DMN that are obscured by conventional network analyses. Crucially, the results challenge the view that intrinsic connectivity rigidly determines function, instead pointing to a dynamic landscape in which task demands flexibly reconfigure how DMN subregions are embedded within large-scale cortical systems.

In conclusion, our findings describe a systematic organizational dissociation within the DMN. Afferent- and efferent-biased subregions exhibit differential patterns of engagement across perception-based and memory-guided cognition. These results link variation in local microarchitecture with large-scale cortical topology and task-related engagement, providing an organizational framework for understanding how association cortex relates to external perception and internal thought.

## 3. Materials and Methods

### 3.1. Participants.

Three independent datasets comprising 259 participants were analyzed. An open-access resting-state fMRI dataset of 40 healthy adults (32) was used to characterize the regional differences in the balance of effective connectivity across the DMN. An additional resting-state sample included 191 participants (age 18 to 31 y, mean ± SD = 20.1 ± 2.25, 68 males), used to examine the intrinsic connectivity profiles of afferent- and efferent-biased DMN regions. To link these subdivisions to cognitive function, 28 undergraduate students were recruited in a task-based fMRI (age 18 to 35 y, mean ± SD = 23.25 ± 4.90, eight males). Three participants were excluded for chance-level behavior, and one for excessive head motion (i.e., relative head motion > 0.2 mm in three runs), leaving 24 participants for fMRI analysis (age 18 to 35 y, mean ± SD = 23.25 ± 4.86, seven males). All were right-handed, native English speakers, with normal or corrected-to-normal vision, and no neurological impairment, learning difficulty, or psychiatric illness. Resting-state dataset 1 was publicly available and used in accordance with the ethical procedures reported in Royer et al. (32). Datasets 2 and 3 were collected by our group with written informed consent and approval from the Research Ethics Committee of the Department of Psychology and York Neuroimaging Centre, University of York (Project P1282/P1291 and Project P1336). All procedures followed the relevant guidelines and regulations, and participants provided informed consent and received monetary reward or course credits.

### 3.2. Materials.

A total of 297 grayscale images were used, comprising 97 faces, 100 scenes, and 100 objects. Face stimuli were selected from the KDEF database (35), and the Radboud Faces Database (47). Only neutral-expressions faces on plain backgrounds were included, and no identity was repeated across trials. Object stimuli were selected from the Bank of Standardized Stimuli [BOSS; (48, 49)], which contains a set of normative high-quality visual stimuli of objects presented on plain backgrounds. Scene images were selected from the Scene UNderstanding (SUN) database (50). To minimize low-level confounds, all images were presented in grayscale. On each trial, participants viewed a pair of images drawn from two different categories, yielding three possible category combinations (Face-Scene, Face-Object, Scene-Object).

### 3.3. Procedure.

We employed an adapted n-back paradigm Fig. 2*A*), in which participants viewed pairs of images drawn from two different categories. On 30% of trials, a colored border cued participants to make a category judgment about one of the two images. Decisions were based either on perceptual information currently on the screen (0-back; perceptually guided) or on their memory of the image presented in the same location on the previous trial (1-back task; memory-guided). Thus, the critical manipulation concerns the source of information available at the decision moment—concurrent sensory input in 0-back trials versus internally maintained information in 1-back trials. In this way, we manipulated both the task (0-back vs. 1-back) and the category (Face vs. Scene vs. Object), yielding six experimental conditions: 0-back face, 0-back scene, 0-back object, 1-back face, 1-back scene, 1-back object. A mixed block-design was used: transitions between 0-back and 1-back trials were unpredictable, requiring participants to maintain information about the previous trial throughout the task. This allowed us to avoid potential confounds from the use of different strategies during encoding in different types of tasks.

Each trial started with a central fixation cross (1,500 to 2,500 ms), followed by either two images within frames (0-back or nonresponse trials) or two empty frames (1-back) presented side-by-side for 2,500 ms. On response trials, one frame turned red, cueing participants to indicate the category of the corresponding image (buttons 1 = face, 2 = scene, 3 = object). On 0-back trials, the relevant image remained visible, so responses were guided by immediate perceptual information. On 1-back trials, the frames contained no images, requiring decision based solely on the memory of previously encoded item. This design ensures that 1-back decisions are temporally decoupled from concurrent sensory input, isolating memory-guided processing. Response trials appeared after 2 to 3 nonresponse trials, with the red box cue presented equally often on the left and right sides throughout the run.

Participants completed four functional runs. Each run comprised 120 trials (18 0-back response trials, 18 1-back response trials, and 84 nonresponse trials), presented in a pseudorandom order. For each type of task, six trials of each category combination were included. Each run lasted 9.6 min, and were separated by a short break, beginning with a 6-s alerting fixation.

### 3.4. Afferent and Efferent Connectivity Map.

The afferent and efferent connectivity maps of the DMN was adopted from Paquola et al. (31), wherein rDCM (36)—a scalable generative model of effective connectivity that allows inferences on the directionality of signal flow, was applied to the resting-state fMRI timeseries of 400 isocortical parcels, covering the entire isocortex, of 40 healthy adults (32). This effective connectivity captures directed interactions among brain regions, with estimates describing how different regions influence each other’s timeseries. In Paquola et al.‘s (31) work, rDCM was applied across the whole cortex, with DMN parcels analyzed as targets to quantify functional input from non-DMN regions (afferent connectivity) and as seeds to quantify functional output to non-DMN regions (efferent connectivity). Here, we specifically computed the contrast between average afferent (DMN parcels as target) and efferent (DMN parcels as seed) to describe regional differences in the balance of effective connectivity within the DMN.

### 3.5. Neuroimaging Data Acquisition.

Structural and functional data were acquired using a 3T GE HDx Excite MRI scanner utilizing an eight-channel phased array head coil at the York Neuroimaging Centre, University of York. Structural MRI was based on a T1-weighted 3D fast spoiled gradient echo sequence [repetition time (TR) = 7.8 s, echo time (TE) = minimum full, FOV = 290 × 290 mm, matrix size = 256 × 256 mm, 176 slices, voxel size = 1.13 mm × 1.13 mm × 1 mm, flip-angle = 90°]. A gradient-echo EPI sequence was used to collect data from 38 bottom–up axial slices aligned with the temporal lobe (TR = 2 s, TE = minimum full, FOV = 192 × 192 mm, matrix size = 64 × 64, slice thickness = 3 mm, slice-gap = 1 mm, and voxel size = 3 mm × 3 mm × 3 mm). Each participant completed four functional runs, each containing 288 volumes. To improve coregistration between structural and functional scans, a fluid-attenuated inversion-recovery (FLAIR) scan was also acquired with the same orientation as the functional scans.

For the resting-state fMRI, a 9-min resting-state fMRI scan was recorded, using single-shot 2D gradient-echo-planar imaging (TR = 3 s, TE = minimum full, flip angle = 90°, matrix size = 64 × 64, 60 slices, voxel size = 3 mm × 3 mm × 3 mm, 180 volumes). Structural scans were based on the same 3D fast spoiled gradient echo sequence (TR = 7.8 s, TE = minimum full, flip angle = 20°, matrix size = 256 × 256 mm, 176 slices, voxel size = 1.13 mm × 1.13 mm × 1 mm). The participants were instructed to focus on a fixation cross with their eyes open and to keep as still as possible, without thinking about anything in particular.

### 3.6. Preprocessing of Task-Based fMRI Data.

All functional and structural data were preprocessed using a standard pipeline and analyzed via the FMRIB (Functional Magnetic Resonance Imaging of the Brain) Software Library (FSL version 6.0, www.fmrib.ox.ac.uk/fsl). Individual FLAIR and T1-weighted structural brain images were extracted using FSL’s Brain Extraction Tool (BET). Structural images were linearly registered to the MNI152 template using FMRIB’s Linear Image Registration Tool (FLIRT). For the functional data, the first three volumes (6-s alerting fixation) were removed to minimize the effects of magnetic saturation, leaving 285 volumes per run. The functional neuroimaging data were analyzed by using FSL’s FMRI Expert Analysis Tool. We applied motion correction using MCFLIRT (51), slice-timing correction using Fourier space time-series phase-shifting (Regular-up), spatial smoothing using a Gaussian kernel of Full Width at Half Maximum (FWHM) 6 mm, and high-pass temporal filtering (sigma = 100 s) to remove temporal signal drift. In addition, motion scrubbing (using the fsl_motion_outliers tool) was applied to exclude volumes that exceeded a framewise displacement threshold of 0.9 mm. One run each from two participants was excluded from the univariate analyses (one due to incorrect button usage, one due to missing data).

### 3.7. Preprocessing of Resting-State fMRI Data.

Preprocessing was conducted in the CONN-fMRI functional connectivity toolbox (CONN), Version 18a (52), based on Statistical Parametric Mapping 12 (http://www.fil.ion.ucl.ac.uk/spm/). Functional data were realigned and unwrapped for motion correction, and potential outlier scans were identified with the Artifact Detection Tool (ART) toolbox. Structural images were segmented into gray matter, white matter, and cerebrospinal fluid tissues, and normalized to the MNI space using the unified segmentation and normalization procedure (53). Functional volumes underwent slice-timing (bottom–up, interleaved) and motion-corrected, skull-stripped and coregistered to the structural image, normalization to MNI space (53), and spatial smoothing with a 6 mm FWHM Gaussian kernel.

CONN automatically generated three first-level covariates for each participant: i) six rigid-body motion parameters from realignment, ii) flagged outliers scans based on ART thresholds (global signal above *z* = 3, subject motion threshold above 0.5 mm, differential motion, and composite motion exceeding 97% percentile in the normative sample), and iii) quality assurance parameters (e.g., the global signal change, the framewise displacement). These, along with white matter and cerebrospinal fluid signals and a rest-effect, were entered as potential confound regressors in the denoising step.

Noise removal was performed using anatomical CompCor approach (54), which removes physiological and motion-related artifacts within a single general linear model (GLM). Functional images were then band-passed filtered (0.01 to 0.1 Hz). A linear detrending term was also applied, eliminating the need for global signal normalization (55, 56). Global signal regression was not performed, consistent with prior work showing that CompCor adequately addresses motion-related and other sources of noise in the BOLD signal (54, 57).

### 3.8. Task-Based fMRI Data Analysis.

The preprocessed time-series data were modeled using a GLM, implemented in FMRIB’s Improved Linear Model (FILM), which corrects for local autocorrelation (58). Task-related regressors were modeled using a double-Gaussian hemodynamic response function. Six explanatory variables (EVs) of interest were defined from response trials to capture decision-related activity: 1) 0-back Face, 2) 0-back Scene, 3) 0-back Object, 4) 1-back Face, 5) 1-back Scene, 6) 1-back Object. A variable epoch model was used, focusing on the response period (response time; from target onset to decision-making), which provides a more physiologically plausible representation of decision-related activity, and improves the statistical power, reliability, and interpretability for results (59). Seven additional EVs of no interest were included: 7) Face-Scene nondecision pairs, 8) Face-Object nondecision pairs, 9) Object-Scene nondecision pairs, and fixation periods following these nondecision stimuli [10) Face-Scene, 11) Face-Object and 12) Scene-Object]. Modeling each nondecision category combination separately allowed us to better characterize encoding and maintenance processes. An additional regressor captured 13) Incorrect Responses across all conditions. The fixation periods following 0-back and 1-back decision trials served as the implicit baseline.

The analysis examined the main effects of Task (0-back vs. 1-back) and Category (Face vs. Scene vs. Object), plus their two-way interaction. The four runs were combined using fixed-effects analyses for each participant. At the group level, contrasts were analyzed using FMRIB’s Local Analysis of Mixed Effects (FLAME1), with automatic outlier deweighting (60). A 50% probabilistic gray-matter mask was applied. Clusters were thresholded using Gaussian random-field theory, with a cluster-forming threshold of *z* = 3.1 and a family-wise-error-corrected significance level of *P* = 0.05.

### 3.9. Gradient Similarity Analysis.

To assess the extent to which each task related to the macroscale gradients of intrinsic connectivity described by Margulies et al. (7), we performed spatial similarity analysis (61). We focused on the first three gradients, which capture the largest variance in resting-state fMRI: Gradient 1, separating sensory-motor cortex from DMN (unimodal–transmodal axis); Gradient 2, distinguishing visual from auditory-motor regions; and Gradient 3, separating control from automatic processing regions. For each participant and each condition, we calculated the spatial correlation (using fsl-cc) between the *z*-stat map of activation (relative to baseline) and each gradient map from Margulies et al. (7), within a cortical mask. Correlation values were Fisher’s R-to-Z transformed and treated as coordinates in gradient space, indexing the similarity between whole-brain task-related activity pattern and intrinsic connectivity gradient.

We then ran three Linear Mixed Models (LMMs) to test how task states (0-back and 1-back) and content type (face vs. object vs. scene) influenced the location of activation along each gradient. In these models, gradient coordinates served as the outcome variable, with task, category, and their interaction as fixed effects. Age and gender were included as nuisance covariates, and ‘participant’ was modeled as a random intercept to account for repeated measures.

LMMs were fitted by restricted maximum-likelihood estimation in R [4.1.1; (62)] using the lme4 package [(1.1.32; (63)]. Significance testing was performed with the lmerTest package [3.1.3; (64)], Using Satterthwaite approximation for degrees of freedom and type 3 sum of squares for *F* tests. Contrasts were set to “contr.sum,” so the intercept reflected the grand mean across all conditions and, for two-level factors, the parameter estimate corresponded to half the difference between levels (65). Estimated marginal means (shown in Fig. 6) were obtained with the emmeans package [1.8.5; (66)]. To control for multiple comparisons, the alpha level for each *F*-statistic was Bonferroni-corrected based on the number of models. Outliers were defined as z-scored gradient coordinates exceeding ±2.5; their z-scores were reset to zero to reduce undue influence. Using this criterion, one outlier was detected along gradient 1, two along gradient 2, and four along gradient 3.

Example model formula: lmer[Z-scored Dimension Coordinate X ~ Task state + Category + Age + Gender + (1 | Participant)]

### 3.10. Analysis of Resting-State fMRI Data.

The resting-state functional connectivity used DMN seeds reflecting distinct afferent and efferent connectivity with non-DMN cortex. At the first-level, whole-brain seed-to-voxel correlations were computed for each seed after preprocessing and denoising of the BOLD timeseries. At the group-level, we contrasted the functional connectivity maps derived from afferent- versus efferent-biased DMN seeds.

### 3.11. Neurosynth Decoding.

Task activation maps were uploaded to Neurovault [https://neurovault.org/collections/13326/; (67)] and decoded using Neurosynth (68), an automated meta-analysis tool that applies text-mining to identify terms from neuroimaging articles that co-occur with reported peak activation. This approach generates terms frequently associated with a spatial map (as in Fig. 3). Results of cognitive decoding were visualized as word clouds using a free online generator (https://www.wordclouds.com/). We manually excluded terms referring to neuroanatomy (e.g., “inferior” or “sulcus”), and repeated terms (e.g., “task” and “tasks”).

## Supplementary Material

Appendix 01 (PDF)

## Data Availability

Neuroimaging data at the group-level are openly available in Neurovault at https://neurovault.org/collections/13326/ (69). Materials and the script for the task are accessible in the Open Science Framework at https://osf.io/9rk6m/ (70). All brain figures were created using BrainNet Viewer [http://www.nitrc.org/projects/bnv/; (71)]. Raw data are not publicly accessible as we do not have sufficient consent from participants. Researchers who wish to access the raw data should contact the Research Ethics and Governance Committee of the York Neuroimaging Centre, University of York, or the corresponding authors. Data will be released to researchers when this is possible under the terms of the GDPR (General Data Protection Regulation).
