# Supplementary material for: Sender–receiver subdivisions of the default mode network in perceptual and memory-guided cognition
Source: Proc Natl Acad Sci U S A. 2026 Apr 7;123(15):e2528851123. doi: 10.1073/pnas.2528851123 (PMC13079981; doi:10.1073/pnas.2528851123)
Supplement: Supplementary file 1 — Appendix 01 (PDF) [file pnas.2528851123.sapp.pdf]

Supplementary Materials

Supplementary Analysis 1: Differential afferent- and efferent-biased DMN connectivity across large-scale networks and cortical gradients

Using an independent resting-state dataset (N = 191), the main analysis showed that efferent-biased DMN regions were more strongly connected to motor and visual systems, whereas afferent-biased regions were preferentially coupled to heteromodal areas (see Figure 1D in the main text). To further quantify these differences, we extracted the mean connectivity strength between the afferent/efferent DMN seeds and each of the Yeo-7 networks for each participant. When calculating DMN-to-DMN connectivity, we excluded the seed voxels themselves (i.e., afferent voxels were excluded when assessing connectivity with DMN, and vice versa) to avoid circularity. Statistical significance was assessed using non-parametric permutation tests. This analysis revealed efferent-biased regions coupled more strongly with sensory-motor, ventral attention, and limbic networks, whereas afferent-biased regions preferentially connected to dorsal attention, frontoparietal, and efferent DMN regions (Supplementary Figure S1A).

To provide the statistical validation for the Gradient 1 effect reported in the main text (Figure 1E), Supplementary Figure S1B presents the corresponding null distributions derived from 10,000 non-parametric permutation tests. These confirm that the observed difference between afferent- and efferent-biased DMN subunits was specific to Gradient 1 ($p < .001$), with no significant effects for Gradients 2 ($p = 1$) or 3 ($p = .10$).

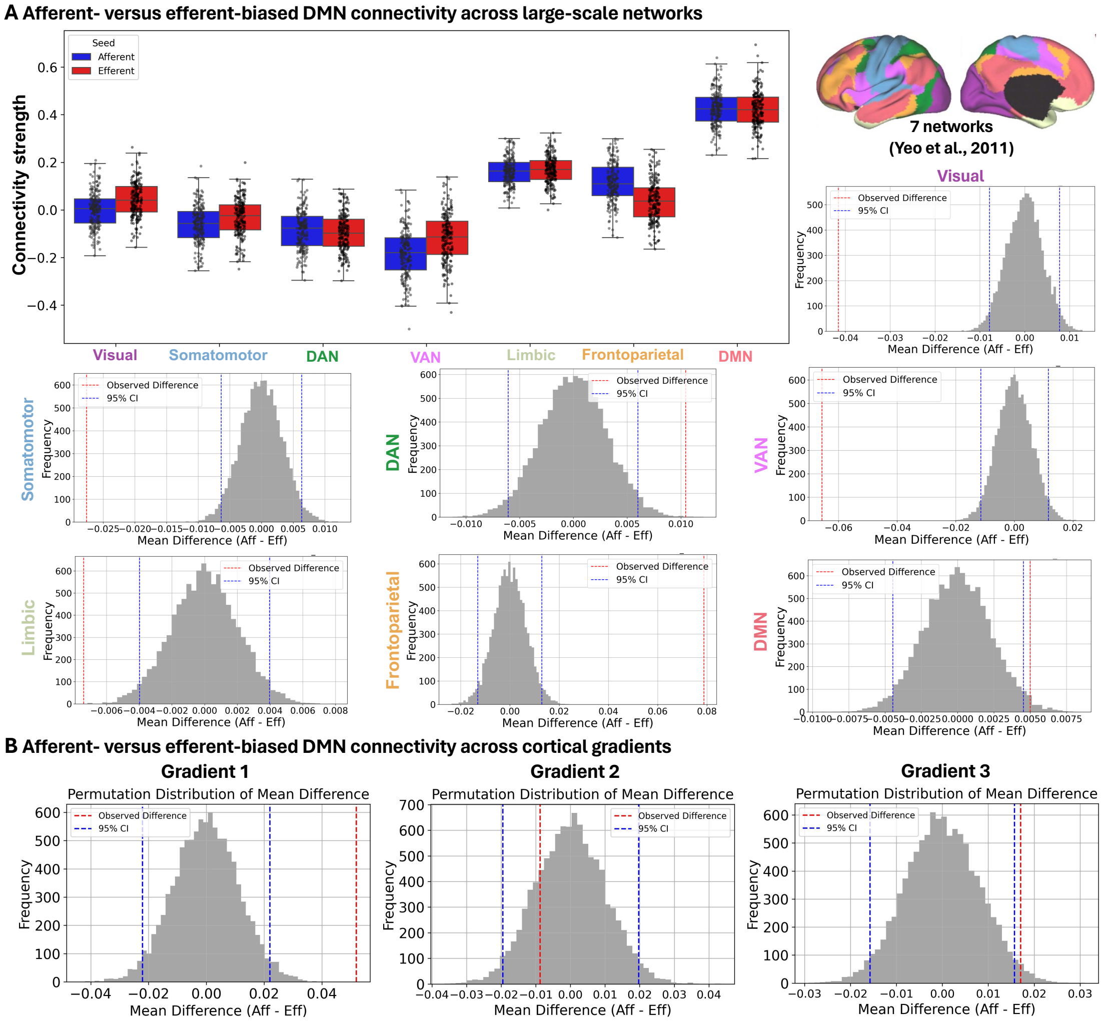

Supplementary Figure S1. **(A)** ***Afferent- versus efferent-biased DMN connectivity differences across large-scale networks.*** Significant differences emerged across all networks: efferent-biased regions coupled more strongly with visual ($p < .001$), motor ($p < .001$), ventral attention ($p < .001$), and limbic networks ($p < .001$), whereas afferent-biased regions were preferentially coupled to dorsal attention ($p < .001$) and frontoparietal ($p < .001$), and efferent DMN regions ($p = .029$; all p-values FDR-corrected). **(B)** ***Null distributions for afferent- and efferent-biased DMN connectivity across cortical gradients.*** Histograms show the frequency distribution of 10,000 non-parametric permutation tests comparing afferent- and efferent-biased connectivity maps to the first three gradients, with seed voxels excluded to avoid bias. The red dashed line indicates the observed difference in similarity between afferent- and efferent-biased maps for the actual data, with positive values reflecting stronger alignment of afferent-biased DMN regions with the corresponding gradient. The two blue lines mark the 75% confidence interval of the null distribution. These results confirm that the effect is specific to Gradient 1, consistent with the main analysis in Figure 1E.

Supplementary Analysis 2: Behavioral effects of perceptual similarity

Behavioral performance in the n-back task was analyzed to assess how perceptual similarity between image pairs influenced reaction time (RT) and accuracy. RT was examined using linear mixed-effects models, and accuracy using generalized linear mixed-effects models

(GLMMs) with a logistic link. RT analyses were restricted to correct responses. All analyses focused on the 0-back and 1-back conditions.

Perceptual similarity between image pairs was quantified using a feature-based representational similarity approach. Each stimulus image was passed through a pretrained ResNet-50 model, and feature vectors were extracted from a mid-to-high level layer that capture visual features relevant for discriminating faces, scenes, and objects. For each trial, similarity was computed as the cosine similarity between the feature vector of target image and non-target image in the n-back task, yielding a continuous similarity value that reflects their perceptual overlap. Higher cosine values indicate greater similarity. These similarity values were then centered across trials.

To control for potential confounds, Trial Number and Category Type were included as covariates: Trial Number accounts for temporal effects such as practice or fatigue, while Category Type accounts for differences across stimulus categories (Faces, Scenes, Objects). RT was modelled using linear mixed-effects models with random intercepts and slopes for similarity by subject:

$$\text{RT or ACC} \sim \text{ImagePairSimilarity}_c \times \text{Task} + \text{Category Type} + \text{TrialNumber} + (1 + \text{ImagePairSimilarity}_c \mid \text{subject})$$

Kenward-Roger approximation was applied to compute denominator degrees of freedom for *F*-tests. Simple slopes of $\text{ImagePairSimilarity}_c$ were estimated for 0-back and 1-back conditions.

For RT, the analysis revealed significant main effects of similarity ($F(1,25.42) = 7.03$, $p = 0.014$), Task ($F(1,1588.35) = 17.99$, $p < 0.001$), Trial Number ($F(1,1607.86) = 133.42$, $p < 0.001$), and Category Type ($F(2,1588.97) = 27.04$, $p < 0.001$). Importantly, there was a significant interaction between similarity and task condition ($F(1,1589.92) = 12.90$, $p < 0.001$), indicating that perceptual similarity influenced RT differently across tasks. Simple slopes showed that similarity was positively associated with RT in the 0-back condition ($\beta = 1.15$, SE = 0.26, $t(101) = 4.41$, $p < 0.001$), with higher similarity resulting in a slower response, but not in the 1-back condition ($\beta = -0.18$, SE = 0.26, $t(101) = -.69$, $p = 0.50$), demonstrating a stronger effect of perceptual similarity on RT in the 0-back task.

For accuracy, the GLMM revealed a significant negative effect of similarity ($\beta = -9.54$, SE = 4.40, *z value* = -2.17, $p = 0.030$), and Task ($\beta = -.87$, SE = 0.21, *z value* = -4.14, $p < 0.001$). Simple-slope analyses showed a significant negative effect of similarity in the 0-back condition ($\beta = -9.54$, SE = 4.40, $p = .030$), indicating that higher perceptual similarity reliably reduced response accuracy. In contrast, the corresponding slope in the 1-back condition was not significant ($\beta = -2.94$, SE = 3.26, $p = .173$), suggesting that accuracy in 1-back trials was not strongly influenced by perceptual similarity. The difference between the two slopes (i.e., Similarity × Task interaction) was not statistically significant ($\beta = 6.60$, SE = 4.85, *z value* = 1.36, $p = .17$).

Together, these RT and accuracy findings provide clear behavioral evidence that 0-back performance is more strongly driven by perceptual similarity than 1-back performance. This dissociation aligns with—and behaviorally validates—the intended cognitive demands of the task design, in which 0-back relies more heavily on perceptual processing while 1-back requires memory retrieval of previously seen items to guide behavior.

Supplementary Analysis 3: Main effects of Category in task activation

To complement the main text, which focuses on Category effects within the 0-back condition, here we report the overall main effects of Category as well as additional Category-related effects observed in 0-back condition.

Face > Scene and Object: Lateral occipital cortex, angular gyrus, and right middle temporal gyrus showed stronger responses during decisions about faces compared to other categories (*Face > Scene/Object*; see Supplementary Figure S2A). Of these face-selective voxels, 65% fell within the DMN (see pie chart in Supplementary Figure S2A). Meta-analysis of this map using Neurosynth revealed that regions linked to face processing associated with terms like "default", "theory mind", and "social" (Supplementary Figure S2A).

Scene > Face and Object: Lateral occipital cortex, precuneus cortex, lingual gyrus, temporal occipital fusiform, and occipital fusiform gyrus showed stronger responses to decisions about scenes relative to other categories (Supplementary Figure S2B). Nearly all voxels within these scene-selective clusters fell within the visual network (see pie chart in Supplementary Figure S2B).

Scene > Face and Object in 0-back: Similarly, 0-back scene targets elicited stronger responses in visual areas—including lateral occipital cortex, precuneus cortex, lingual gyrus, temporal occipital fusiform, and occipital fusiform gyrus—compared to the other two types of category targets (Supplementary Figure S2C). Again, 97% of these clusters fell within the visual network (see pie chart in Supplementary Figure S2C).

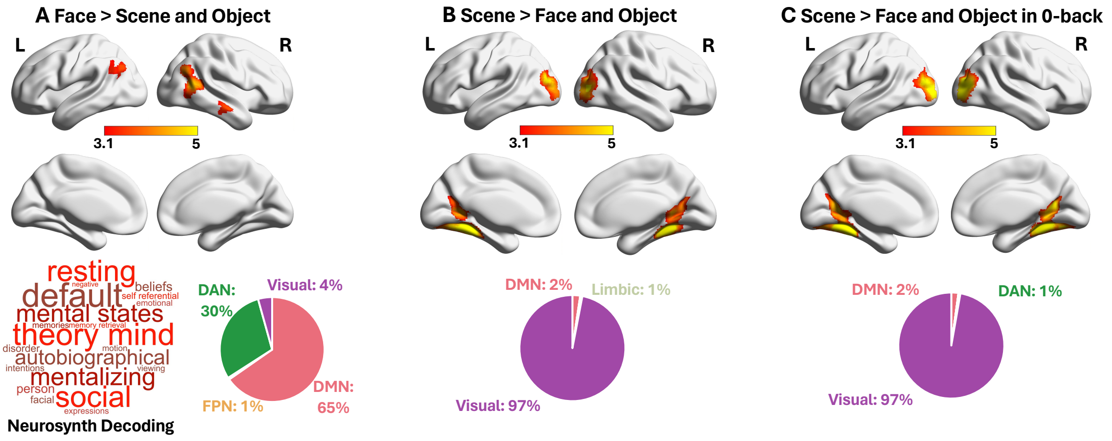

Supplementary Figure S2. **(A)** Regions showing greater activity for face-category decisions (*Face > Scene/Object*), combining both tasks. Many voxels overlapped with the DMN. Using Neurosynth, a meta-analysis of face-sensitive regions revealed strong associations with the functional terms of "default", "theory mind", and "social". **(B)** Regions showing stronger activation for scene-category decisions (*Scene > Face/Object)*, combining both tasks. These clusters largely fell within the visual network. **(C)** Regions showing stronger activation for scene-category decisions on 0-back trials (*Scene > Face/Object in 0-back*). These clusters also largely fell within the visual network. All maps are thresholded at $z > 3.1$ ($p < .05$). L = Left hemisphere; R = Right hemisphere.

Supplementary Analysis 4: A dissociation within DMN for perceptually grounded and memory-guided cognition

To further examine the degree to which perceptually-decoupled state and face perception showed a clean dissociation within DMN, we used the DMN clusters identified in the contrasts of *1-back > 0-back* and *Face > Scene/Object in 0-back*, shown in Figure S3, as masks and extracted the mean % signal change for each experimental condition for each participant. A 2 (Task: 0-back vs. 1-back) by 3 (Category: Face vs. Scene vs. Object) repeated-measures ANOVA was performed for each DMN map.

For the 1-back DMN map, only the main effect of Task was significant, $F(1,23) = 22.46$, $p < .001$, $\eta_p^2 = .49$, suggesting a stronger activation during the 1-back task relative to the 0-back task. There were no significant effects of Category, $F(2,46) = .37$, $p = .69$, $\eta_p^2 = .02$, or interaction, $F(2,46) = 1.24$, $p = .30$, $\eta_p^2 = .05$. For the Face perception DMN map, only the main effect of Category was significant, $F(2,46) = 15.13$, $p < .001$, $\eta_p^2 = .40$, suggesting a stronger response to faces compared to the other two categories (Face vs. Scene: $t(23) = 5.21$, Holm-Bonferroni corrected $p < .001$; Face vs. Object: $t(23) = 4.58$, Holm-Bonferroni corrected $p < .001$; Scene vs. Object: $t(23) = .82$, Holm-Bonferroni corrected $p = .41$). There were no significant effects of Task, $F(1,23) = .82$, $p = .38$, $\eta_p^2 = .03$, or interaction, $F(2,46) = 1.85$, $p = .17$, $\eta_p^2 = .07$. These results are presented in Supplementary Figure S3.

In summary, these results indicate a clear dissociation within the DMN: memory-guided decision-making (1-back task) selectively engages the DMN clusters associated with task demands, whereas face perception selectively engages the DMN clusters associated with perceptually grounded processing.

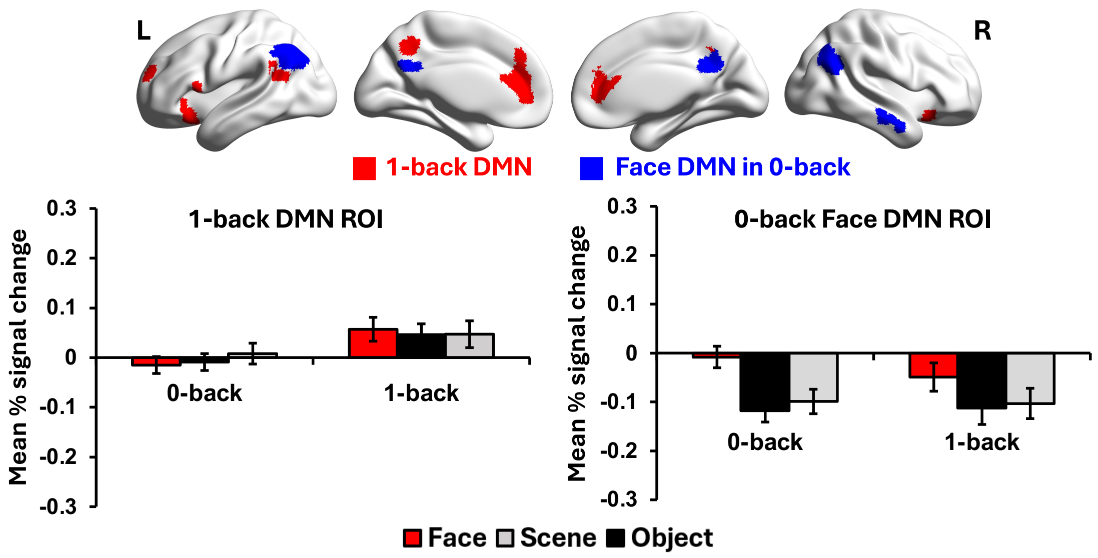

Supplementary Figure S3. Mean % signal change in memory-guided and perception-grounded DMN maps. The bar charts plot the mean percentage signal change in the *1-back > 0-back* (in red) and *Face > Scene and Object in 0-back (in blue)* DMN maps for each experimental condition relative to implicit baseline (i.e., the fixation interval after 0-back and 1-back decision trials). Error bars depict the standard error of the mean. L = Left hemisphere; R = Right hemisphere.

Supplementary Analysis 5: Task activation in DMN subsystems

To examine how the dissociable DMN effects relate to canonical functional subdivisions, we mapped each DMN voxel associated with memory-guided cognition (*1-back > 0-back*) and face perception (*Faces versus Others in 0-back*) within the dorsomedial, core and medial temporal DMN subsystems, as defined by Yeo et al. (2011) in their finer-grained parcellation of intrinsic connectivity into 17 cortical networks. The results are presented in Supplementary Figure S4, where different colors represent distinct DMN subsystems. DMN regions engaged during perceptually-decoupled 1-back decision-making fell within the core (blue; 64%) and dorsomedial (red; 36%) DMN subsystems, while the majority of DMN voxels showing greater activation to face perception fell within the core DMN (blue; 89%).

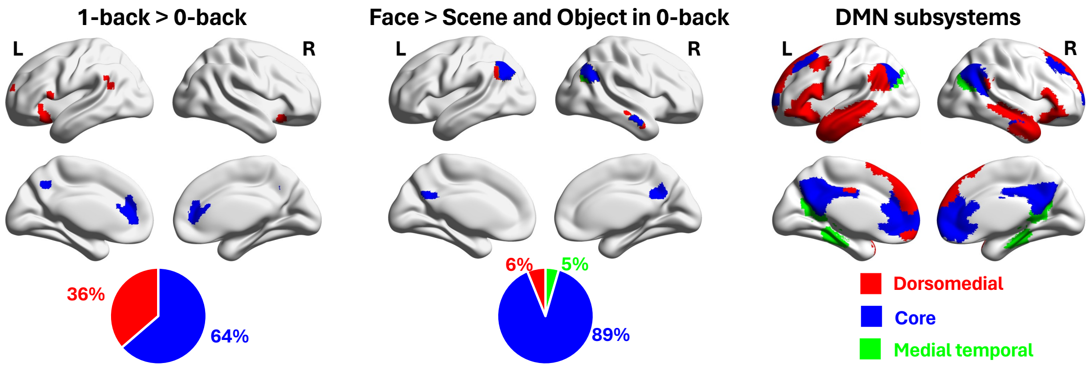

Supplementary Figure S4. Distinctive activation in DMN subsystems. Relationship between observed activity during perceptually-decoupled decision-making (i.e., *1-back > 0-back*) and face perception (i.e., *Face > Scene/Object in 0-back*) and the subsystems of the DMN as described by Yeo et al. (2011) in a 17-network parcellation of intrinsic funcitonal connectivity. Regions in red correspond to the dorsomedial subsystem, blue to the core subsystem, and green to medial temporal subsystem. Pie charts show the proportion of significant voxels associated with each activity pattern that fell within each subsystem. L = Left hemisphere; R = Right hemisphere.

Supplementary Analysis 6: Category-selective peak activation in visual cortex

To complement the main-text results highlighting category-selective responses in visual regions, we provide detailed peak activation maps for each category across the visual cortex (Supplementary Figures S5).

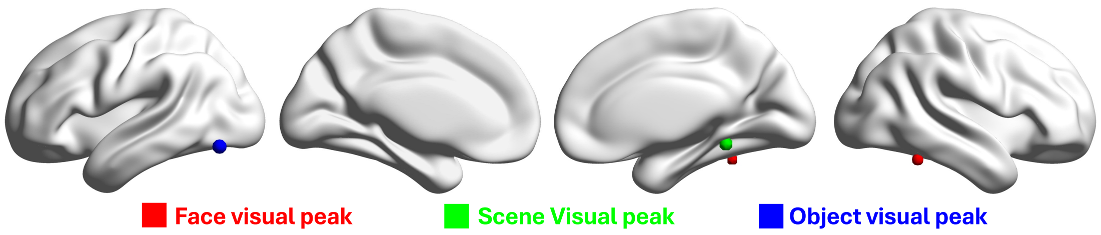

Supplementary Figure S5. Category-selective peak activation in visual cortex. Peak activation sites in visual cortex are shown for each category: scene (MNI coordinates: 28, -44, -12; Parahippocampal Place Area, PPA), face (MNI coordinates: 42, -48, -22; Fusiform Face Area;

FFA), and object (MNI coordinates: -48, -72, -14; Lateral Occipital Cortex, LOC).

<u>Supplementary Analysis 7: Whole-brain interaction between Task and Category</u>

To complement the main text, which focuses on the Task effects and Category effects within the 0-back condition, we additionally report the interaction between these two factors. A significant interaction between Task (0-back vs. 1-back) and Category (Face vs. Scene vs. Object) was observed in lateral occipital cortex and temporal occipital fusiform (see Supplementary Figure S6). To understand the nature of this effect, we extracted parameter estimates for each condition in each cluster. In the 0-back task, these regions showed the strongest activation to scene decisions, followed by object decisions, and the weakest activation to face decisions (Lateral occipital cortex: Face vs. Scene: $t(23) = -10.88$, $p < .001$; Face vs. Object: $t(23) = -8.11$, $p < .001$; Object vs. Scene: $t(23) = -5.13$, $p < .001$; Temporal occipital fusiform: Face vs. Scene: $t(23) = -10.64$, $p < .001$; Object vs. Scene: $t(23) = -6.89$, $p < .001$; Face vs. Object: $t(23) = -6.44$, $p < .001$; all Holm-Bonferroni corrected for the number of comparisons per site). There was no significant difference in visual activation during the 1-back task. These results show that the responsiveness of visual cortex to perceptual inputs in the 0-back task was modulated by category information: while face perception elicited more activation in DMN, scene perception was associated with stronger visual activation.

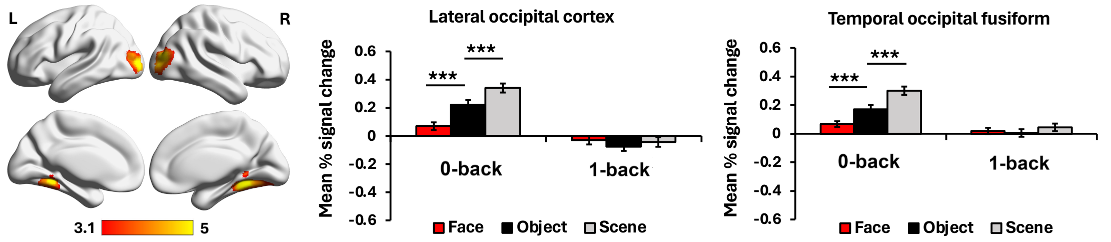

Supplementary Figure S6. Interaction effect in visual cortex. Activation showing Task (0-back vs. 1-back) by Category (Face vs. Scene vs. Object) interaction in visual cortex. Bar charts plot the mean percentage (%) signal change in the visual clusters for each condition relative to implicit baseline (i.e., the fixation interval after 0-back and 1-back trials), revealing differences in activity across different categories in the 0-back task, with little response in the 1-back task. Error bars depict the standard error of the mean. All maps are thresholded at $z > 3.1$ ($p < .05$). L = Left hemisphere; R = Right hemisphere.

<u>Supplementary Analysis 8: Spin tests of the spatial location of DMN subsystems</u>

To determine whether the canonical DMN subsystems were consistently situated at the transmodal end of the principal gradient (unimodal-transmodal axis), we examined the Core, Dorsomedial, and Medial Temporal subsystems as defined by Yeo, et al. (28) 17-network parcellation. Notably, not all subsystems showed this pattern. The Core and Dorsomedial DMN subsystems were significantly transmodal ($p < .001$ for both), whereas the Medial Temporal subsystem did not differ from chance ($p = .71$; Bonferroni corrected; Supplementary Figure S7). This indicates that transmodal engagement is uneven across DMN subsystems, with Core and Dorsomedial components contributing most strongly, while the Medial Temporal system shows a distinct profile.

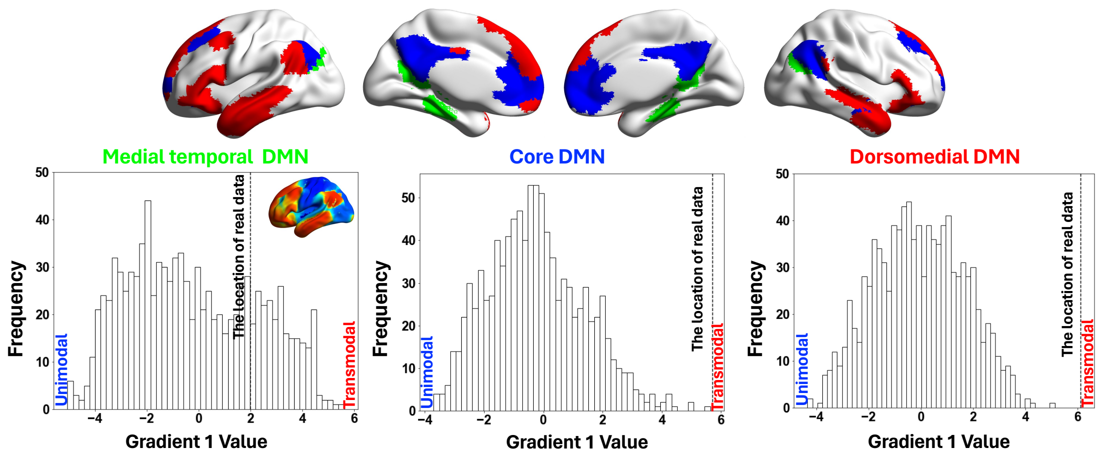

***Supplementary Figure S7.* Spin tests of the canonical DMN subsystems' locations.** The null distributions for medial temporal, core, and dorsomedial DMN subsystems. Gradient 1 (top left) separates transmodal (red) from unimodal cortex (blue). Histograms depict the frequency distribution of 1000 spin permutations, with the dashed line indicating the observed location of the actual data on Gradient 1. These results demonstrate that only core and dorsomedial DMN regions occupy the transmodal apex significantly beyond chance.

Supplementary Analysis 9: Relationship of whole-brain gradients to whole-brain activity patterns

To complement the main text, which focuses on Task (1-back vs. 0-back) and Category effects in 0-back, here we report the omnibus ANOVA including the Task × Category interaction and tests of simple effects contrasting all categories.

The mixed models comparing the coordinates of each task map along the three state-space gradients revealed significant differences between 0-back and 1-back states [Gradient 1: $F(1,115) = 6.39$, $p = .013$; Gradient 2: $F(1,115) = 175.53$, $p < .001$; Gradient 3: $F(1,115) = 21.78$, $p < .001$]. Along Gradient 1 (unimodal–transmodal axis), 0-back states were located further towards the sensory-motor end than 1-back states [$b = -.22$, 95% CI [-.39, -.05], $t(115) = -2.53$, $p = .013$]. Along Gradient 2 (visual-to-motor axis), 1-back states were located further from the visual end than 0-back states [$b = 1.03$, 95% CI [0.87, 1.18], $t(115) = 13.25$, $p < .001$]. Along Gradient 3 (control–automatic axis), 1-back task was further at the control end of this dimension than 0-back task [$b = -.44$, 95% CI [-.63, -.26], $t(115) = -4.67$, $p < .001$].

Category effects were significant along Gradient 1, $F(2,115) = 10.71$, $p < .001$, and Gradient 2, $F(2,115) = 4.25$, $p = .017$, but not Gradient 3, $F(1,115) = 2.07$, $p = .13$. On Gradient 1, face states were further toward the association end than object [$b = .42$, 95% CI [.16, .67], $t(115) = 3.97$, $p < .001$] and scene states [$b = .42$, 95% CI [.17, .68], $t(115) = 4.04$, $p < .001$], consistent with the DMN response that was observed for this category. On Gradient 2, scene states were located further towards the visual end than face states [$b = .27$, 95% CI [.04, .50], $t(115) = 2.88$, $p = .014$].

Finally, Task × Category interactions were observed on Gradient 1, $F(2,115) = 4.49$, $p = .013$, and Gradient 2, $F(2,115) = 5.84$, $p = .004$, but not Gradient 3, $F(2,115) = .43$, $p = .65$. On Gradient 1, object states differed between 0-back and 1-back tasks [$b = -.52$, 95% CI [-.88, -.16], $t(115) = -3.51$, $p = .002$], being further towards the association end in the 1-back condition, but not face states [$b = .11$, 95% CI [-.25, .47], $t(115) = .72$, $p = 1.000$] or scene

states [b = -.24, 95% CI [-.60, .12], t(115) = -1.59, p = .34]. Furthermore, in 0-back, face states were further towards the association end than both object [b = .73, 95% CI [.33, 1.13], t(115) = 4.93, $p < .001$] and scene states [b = .60, 95% CI [.20, .99], t(115) = 4.02, p = .001]. In contrast, in the 1-back condition, face states were not significantly different to object states [b = .10, 95% CI [-.30, .50], t(115) = .69, p = 1.000] and scene states [b = .25, 95% CI [-.15, .65], t(115) = 1.70, p = .553] , but no differences emerged in 1-back. On Gradient 2, scene states in 0-back were further towards the visual end than face states [b = -.58, 95% CI [-.94, -.22], t(115) = -4.29, p = .001], whereas no differences were found in the 1-back.

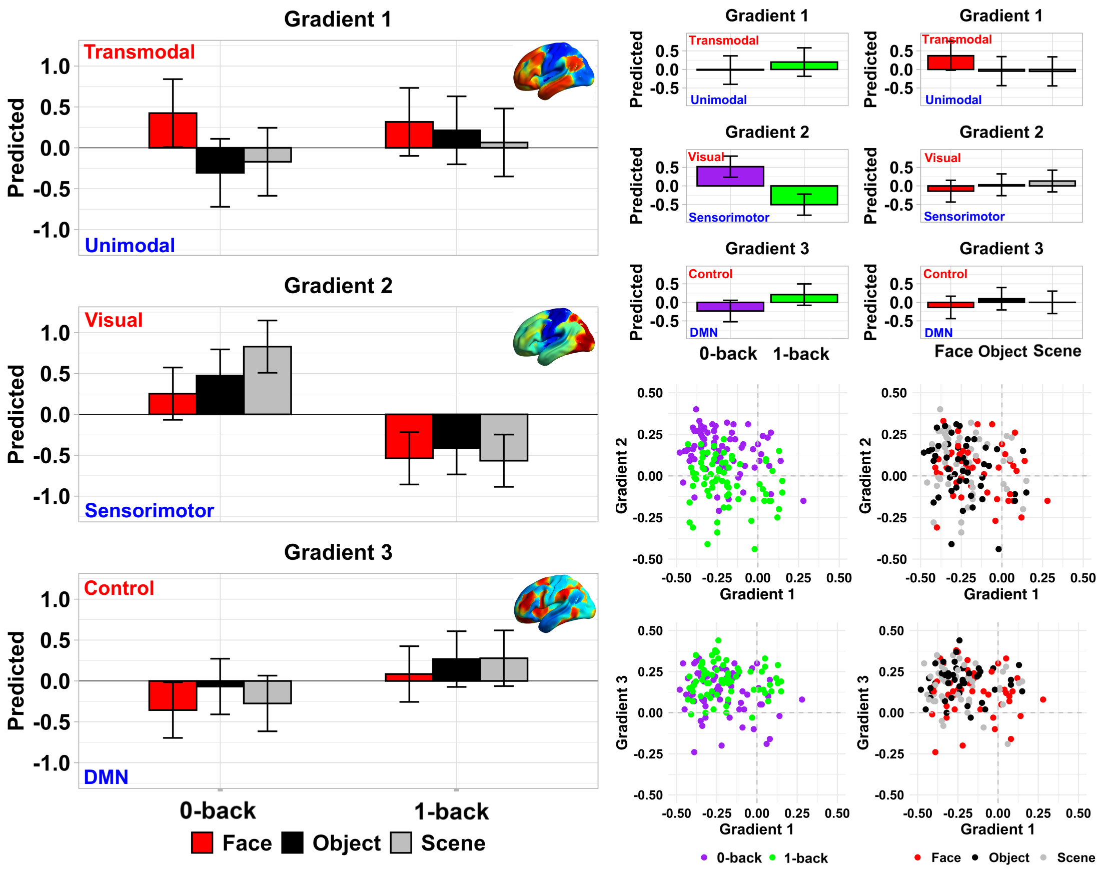

Supplementary Figure S8. Results of gradient similarity analyses. Spatial similarity of the neural response of each experimental condition with intrinsic gradients of brain organisation (Margulies et al., 2016). Bar charts (left) show the similarity along the principal gradient (i.e., transmodal-unimodal; top), the visual–motor gradient (middle), and the control-DMN gradient (bottom). Bar charts (upper right) shows the differences by task (*1-back vs. 0-back)* and category (*Face vs. Object vs. Scene)*, with y-axis showing the estimated marginal means (i.e., predicted means). The error bars on the graphs are 95% confidence intervals of the predicted means. Scatter plots (lower right) show the distribution of raw data, with each point representing a single condition-specific activation map.
